# Targeting Dectin-1 and or VISTA enhances anti-tumor immunity in melanoma but not colorectal cancer model

**DOI:** 10.1007/s13402-024-00950-w

**Published:** 2024-04-26

**Authors:** Siavash Mashhouri, Amirhossein Rahmati, Ako Azimi, Roy A. Fava, Ismail Hassan Ismail, John Walker, Shokrollah Elahi

**Affiliations:** 1 Department of Dentistry, Division of Foundational Sciences, Faculty of Medicine and Dentistry, University of Alberta, Edmonton, Canada; 2 Department of Veterans Affairs Medical Center, Research Service, White River Junction, VT USA; 3 Department of Medicine, Geisel School of Medicine at Dartmouth, Dartmouth Hitchcock Medical Center, Lebanon, NH USA; 4 Department of Oncology, Faculty of Medicine and Dentistry, University of Alberta, Edmonton, Canada; 5 Li Ka Shing Institute of Virology, University of Alberta, Edmonton, Canada; 6 Biophysics Department, Faculty of Science, Cairo University, Giza, 12613 Egypt

**Keywords:** Dectin-1+ T cells, VISTA KO mice, Dectin-1 KO mice, Dectin-1+ Myeloid cells, PD-L1+Dectin-1+ myeloid cells

## Abstract

**Purpose:**

Acquired resistance to immune checkpoint blockers (ICBs) is a major barrier in cancer treatment, emphasizing the need for innovative strategies. Dectin-1 (gene *Clec7a*) is a C-type lectin receptor best known for its ability to recognize β-glucan-rich structures in fungal cell walls. While Dectin-1 is expressed in myeloid cells and tumor cells, its significance in cancer remains the subject of controversy.

**Methods:**

Using Celc7a-/- mice and curdlan administration to stimulate Dectin-1 signaling, we explored its impact. VISTA KO mice were employed to assess VISTA’s role, and bulk RNAseq analyzed curdlan effects on neutrophils.

**Results:**

Our findings reveal myeloid cells as primary Dectin-1 expressing cells in the tumor microenvironment (TME), displaying an activated phenotype. Strong Dectin-1 co-expression/co-localization with VISTA and PD-L1 in TME myeloid cells was observed. While Dectin-1 deletion lacked protective effects, curdlan stimulation significantly curtailed B16-F10 tumor progression. RNAseq and pathway analyses supported curdlan’s role in triggering a cascade of events leading to increased production of pro-inflammatory mediators, potentially resulting in the recruitment and activation of immune cells. Moreover, we identified a heterogeneous subset of Dectin-1+ effector T cells in the TME. Similar to mice, human myeloid cells are the prominent cells expressing Dectin-1 in cancer patients.

**Conclusion:**

Our study proposes Dectin-1 as a potential adjunctive target with ICBs, orchestrating a comprehensive engagement of innate and adaptive immune responses in melanoma. This innovative approach holds promise for overcoming acquired resistance to ICBs in cancer treatment, offering avenues for further exploration and development.

**Supplementary Information:**

The online version contains supplementary material available at 10.1007/s13402-024-00950-w.

## Introduction

During the past decade, the field of immuno-oncology has dramatically advanced. Variety of immunotherapy approaches are introduced to the clinic to enhance durable immunity against solid tumours. However, high immunotoxicity and low immunogenicity against different types of cancer limited their clinical applications. Therefore, targeting appropriate pathways within the tumor microenvironment (TME) to induce the least toxicity with the highest efficacy is the main focus of immunotherapy. This will allow the design of novel therapies according to the patient’s specific features and liberates us from common monotherapy [1, 2]. Although CD8^+^ T cells appear to be the main players against tumor cells, their effector functions can be impacted by the immunological properties of other immune cells (e.g. antigen-presenting cells (APCs)) and non-immune cells [3, 4]. In recent years, the role of other innate immune cells such as macrophages and neutrophils has received substantial attention in the cancer field [5]. For example, anti-inflammatory macrophages (M2) can modulate the TME milieu in the favor of tumor cells [6]. Hence, targeting macrophages via the pattern recognition receptors (PRRs) with cognate agonists combined with immune checkpoint inhibitors (ICIs) is a novel approach proposed for solid tumours [7–9]. The PRRs are integral components of innate immunity in maintaining tissue homeostasis and responding to intruding pathogens [10, 11]. For many years, PRRs were represented by Toll-like receptors (TLRs) and studies on the roles of PRRs in cancer therapy were mainly limited to a few members of the TLR family [12, 13]. However, recent studies have shown that the role of PRRs in tumour immunity extends to other PRR families [9]. Among them, C-type lectin receptors (CLRs) are a large family of soluble and transmembrane proteins that comprise a minimum of one conserved carbohydrate-recognition domain binding to glycan structures in a Ca2^+^ dependent manner [14]. Not all CLRs but only a subgroup of CLRs such as Dectin-1, Dectin-2, MINCLE and DC-SIGN are considered as PRRs due to their ability to recognize pathogen-associated molecular patterns (PAMPs) and damage-associated molecular patterns (DAMPs) to initiate innate immune signalling pathways [15]. The pathogen-recognizing CLRs are mainly expressed by myeloid cells and play essential roles in regulating innate immunity against fungal infections [16]. Recently, it has been shown that CLRs are also able to bind to non-carbohydrate molecules such as protein and lipid ligands [17–19]. Dendritic cell-associated C-type lectin-1 (Dectin-1) is one of the best-described CLR molecules that acts as a PRR [20]. A full-length Dectin-1, also known as isomer A, comprises one extracellular C-type lectin-like domain, stalk region, a transmembrane domain and immunoreceptor tyrosine-based activation motif (ITAM) [21, 22]. Dectin-1 differentially interacts with β-glucan structures, proteins, chitin, mannans and lipids on pathogens and tumour cells [23]. Because of the broad range of interactions with various ligands, it is expected that Dectin-1 contributes to immune homeostasis under physiological and pathological conditions [24].

A few studies have shown that stimulating Dectin-1 by cognate agonists like curdlan, zymosan and yeast-derived β-glucans boosts anti-tumour immunity. For instance, NK cell-dependent tumour cell clearance relies on Dectin-1 interaction with N-glycan structures of tumour cells through activation of the IRF5 transcription factor and NK-mediated tumour cell killing [25]. Another study reported that activation of CARD9 axis following Dectin-1 and β-glucans ligation resulted in macrophage metabolic reprograming and M1 polarization of tumour associated-macrophages [26]. This study also showed that oral administration of β-glucans to tumour-bearing mice reduced tumour burden and enhanced effector T cell activation. In contrast, it is reported that activation of Dectin-1 and other members of the CLR family such as Mannose Receptor (CD206) results in tumour progression and an immunosuppressive phenotype in the TME [17, 27, 28]. Similarly, the interaction of Dectin-1 with Galectin-9 (Gal-9) and annexin molecules in TME results in detrimental outcomes in a pancreatic ductal adenocarcinoma (PDA) mouse model [17, 28]. Despite that Dectin-1 signalling pathway leads to pro-inflammatory responses under physiological conditions, the ultimate effect of Dectin-1 activation depends on the type of interacting ligands and tissue microenvironment.

In the present study, we investigated the distribution of Dectin-1 expression in the TME, spleen and peripheral blood of two tumour models; B16-F10 melanoma and CT26 colorectal cancer. We determined that overall expression of Dectin-1 was remarkably high in tumor and myeloid cells in the TME of both immunogenic and non-immunogenic tumour models. However, it was more remarkable in the non-immunogenic model, B16-F10 melanoma model. We also characterized the tumor resident myeloid cells, tumor infiltrating lymphocytes (TILs) for the expression of Dectin-1, and analyzed the effector functions of Dectin-1^+^ versus Dectin-1- myeloid cells in both models. Given a significant co-expression of VISTA, PD-L1, PD-L2 and TIM-3 in myeloid cells, enabled us to discover the co-localization of Dectin-1 with VISTA and PD-L1 in the TME. To better characterize the role of Dectin-1 in vivo, we compared Dectin-1 deletion (using Dectin-1 knock-out (DKO) versus Dectin-1 stimulation (curdlan-treatment) of wild-type mice. Given the co-expression/co-localization of Dectin-1 with VISTA, we further investigated the role of VISTA in DKO mice and similarly analyzed targeting Dectin-1 with and without curdlan in the B16-F10 model. Taken together, these results imply that Dectin-1 stimulation should be considered in combination with ICBs to enhance both arms of immune system against cancer.

## Methods and materials

### Ethics statement

All animal studies were carried out according to the Guide for the Care and Use of Laboratory Animals of the Canadian Council for Animal Care. The experimental protocol was approved by the Animal ethics boards at the University of Alberta (Protocol # AUP00002737). Similarly, human studies were approved by the human ethics board at the University of Alberta (Pro00063463). Written consent form was obtained before the blood draw.

### Animals

Male and female BALB/c and C57BL/6 mice were purchased from the Charles River Institute. VISTA^-/-^ mice in C57BL6/J background were kindly provided by Dr. Roy A. Fava [29]. Dectin-1 knockout (clec7a^-/-^) mice were purchased from the Jackson laboratory. All animals were housed under controlled environmental conditions within the animal care facility at the University of Alberta. We also used age-sex-matched mice (6–8 weeks) in all of our experiments.

### Human peripheral blood processing

Human peripheral blood mononuclear cells (PBMCs) were isolated using Ficoll®-Paque Premium (GE, Chicago, IL, USA) from fresh blood samples according to our protocols [30, 31]. PBMCs were resuspended in RPMI 1640 supplemented with 10% FBS and antibiotics (100 U/L Penicillin, 100 mg/mL Streptomycin). Fresh PBMCs were used for flow cytometry analysis.

### Animal cancer models

B16-F10 melanoma and CT26 colorectal tumour cells, originally obtained from the ATCC, were injected subcutaneously on the left flank of mice under anesthesia conditions. Cell numbers were based on our previous experiments (B16-F10 = 1 × 10^5^ cells, CT26 = 1 × 10^5^ cells). Following 5–7 days after injection, palpable tumours formed. For immunotherapy experiments, animals received intraperitoneally (i.p.) curdlan (15 mg/Kg, Sigma-Aldrich curdlan from Alcaligenes faecalis) or anti-VISTA antibody (250 μg/mouse, Bio X cell 13F3) alone or a combination. Dissolved curdlan was further diluted in PBS prior to injection, and control animals received the same volume of the vehicle in the same manner. In the case of anti-VISTA immunotherapy, the antibody was administered five days after tumour challenge every three days for 10 days (three injections) and controls received the Armenian hamster IgG isotype control (Cat#BE0091, Bio X cell). In the case of curdlan immunotherapy, curdlan was injected a day after tumour challenge every two days for 10 days (five injections). Treatments were determined based on previous reports [32]. Animals were kept until two days after the last injection and tumour tissues and spleens were harvested for further analysis. Tumor size calculated according to the length × width^2^/2 formula, where length represents the largest diameter of tumor and width represents the perpendicular tumor diameter.

### Tissue processing and cell isolation

To obtain a single-cell suspension, spleens were ground between sterile frosted glass slides in 7 ml of 1 × RBC lysis buffer before mixing with 7 ml RPMI culture media and then filtered through nylon mesh as reported elsewhere [33]. Then cells were resuspended in RPMI 1640 Medium (Sigma) with 1% penicillin/streptomycin (Sigma), 10% FBS (Sigma), and nonessential amino acids (Sigma). Tumour tissues were dissected aseptically, washed with Hanks’ Balanced Salt Solution two times, cut in small pieces by scalpel in lysis buffer (DNase [20 U] and Collagenase type IV [2000 U]). Tumour samples were then transferred to 15 ml conical tubes and incubated for 25 mins at 37 °C inside a shaking incubator. Tumour samples were washed by RPMI 1640 Medium and filtered through 70 and 40 μm nylon mesh. To exclude excess tissues and debris, samples were centrifuged on Ficoll®-Paque PREMIUM 1.084. Finally, isolated cells were washed and prepared for further analysis.

### Flow cytometry, image cytometry and cell sorting

The fluorochrome-conjugated antibodies were purchased from ThermoFisher Scientific, BD Biosciences, or BioLegend (Supplementary Table 1). Also, antibodies, such as CD14 (M5E2), CD11c (B-Iy6), and Dectin-1 (15E2) were used on human samples. The LIVE/DEAD kit (Life Technologies) was used to exclude dead cells. For quantifying cytokines and cytolytic molecules we performed intracellular cytokine staining (ICS) according to our protocols [34, 35]. Stained cells were fixed in paraformaldehyde (PFA, 4%), and data were acquired on a Fortessa-X20 or LSR Fortessa-SORP flow cytometer (BD Biosciences) or Amnis® ImageStream®X Mk II Imaging Flow Cytometer. Data were analyzed using Flow Jo software (version 10.7) and IDEAS. For cell sorting, stained cells were resuspended in FACS buffer and sorting was performed on a SONY MA900 Multi-Application Cell Sorter.

### Gene expression assay

Bulk tumour tissues or spleens were subjected to total RNA extraction in TRIZOL reagent (Invitrogen) using the RNeasy kit (Qiagen, Venlo, The Netherlands). A Nano-Drop ND-1000 Spectrophotometer (NanoDrop Technologies, Wilmington, DE, USA) was used to check the quantity and quality of RNA for each sample. At least 500 ng of the isolated RNA was reverse transcribed employing a QuantiTect Reverse Transcription kit (Qiagen). Gene expression of clec7a was calculated by the 2−ΔΔCt method according to our protocols [36, 37]. Glyceraldehyde phosphatidyl hydrogenase (GAPDH) was used as a housekeeping gene to normalize the cDNA levels. The negative controls contained water or reverse-transcription negative RNA instead of template DNA.

### Immunofluorescent staining (IF)

Following tumour dissection, tumour tissues were rinsed with PBS and fixed in 4% paraformaldehyde overnight. Fixed tissues were subjected to sectioning at the University of Alberta HistoCore Center. Fixed tissues then dehydrated in ethanol series, rinsed with xylene and embedded in paraffin [38]. Paraffin-embedded tissues were cut on a microtome and collected on slides. The paraffin slides were checked under the light microscope, and high-quality ones were deparaffinized by washing twice in xylene for 10 min, twice in 100% EtOH for 10 min, one wash in 95 % EtOH for 5 min, one wash in 70% EtOH for 5 min, and finally a 5-min wash in H2O. Next, slides were incubated in pre-warmed citrate buffer pH 6.0 for 10 min in a water bath at 92 °C. Deparaffinized slides were removed and allowed to cool to room temperature and then washed three times with 1× PBS in 5 min intervals. To minimize the binding of non-specific antibodies, slides were incubated in 10% donkey serum in PBST at room temperature for 1 h and washed three times with 1x PBS in 5 min intervals. For staining of CD11b and Dectin-1, samples were incubated in 100 µL of CD11b polyclonal primary antibody (1:200 InvitroGen PA5-79532) and 100 µL of Dectin-1 primary antibody (1:200 InvitroGen MA5-16477) overnight in a moist chamber at 4 °C, respectively. For the negative control, samples were incubated with 100 µL PBS overnight in a moist chamber at 4 °C. The next day slides were washed three times with 1× PBS in 5 min intervals. Then, the slide was stained in the dark with the secondary antibody either 100 µL of Alexa Flour 488 secondary (1:1000 Invitrogen A32790) or Alexa Flour 555 (1:1000 Invitrogen A48263). The slides were incubated 1 hour in the dark at room temperature and then washed three times with 1× PBS for 5 min each wash. Slides were then incubated with 100 µL of DAPI (1:1000 Invitrogen D1306) for 10 minutes and washed three times with 1× PBS in 5 min intervals. A drop of ProLong Mountant (Invitrogen P36934) was added to each sample before coverslips were placed.

### Bulk-RNAseq analysis

We re-analyzed a published dataset (GSE148850) in which BM-neutrophils from wild type mice were either treated with curdlan (100 g/ml) for 3 hrs or untreated before RNA extraction. Pseudo-alignments against the mouse transcriptome (ensembl_GRCm38) were carried out using Kallisto with bias correction and 100 bootstraps [39]. RNAseq data was evaluated at the transcript as well as the gene level where transcript expression was consolidated using tximport [36]. Differential expression (DE) analysis was conducted using count data with the DESeq2 R package (R version 4.2.0) [40]. Differentially expressed genes demonstrated corrected *P*-value (*P*_adj_) < 0.05 and −1 < log_2_ fold change (FC) > +1. Heat map were generated using R scripts. Bubble plots were generated using Matplotlib in Python.

### Statistical analysis

Statistical comparison between two groups was performed by nonparametric t-test. Multiple comparisons between groups (>2) were conducted by the Kruskal-Wallis test, followed by Dunn’s multiple comparison correction in Prism 9.2.0 (283). Correlation between variables calculated using nonparametric spearmen correlation analysis. Results are expressed as mean ± SEM. *P*-value < 0.05 was considered as statistically significant.

## Results

### Dectin-1 is strongly expressed in a non-immunogenic tumour

**Fig. 1:**
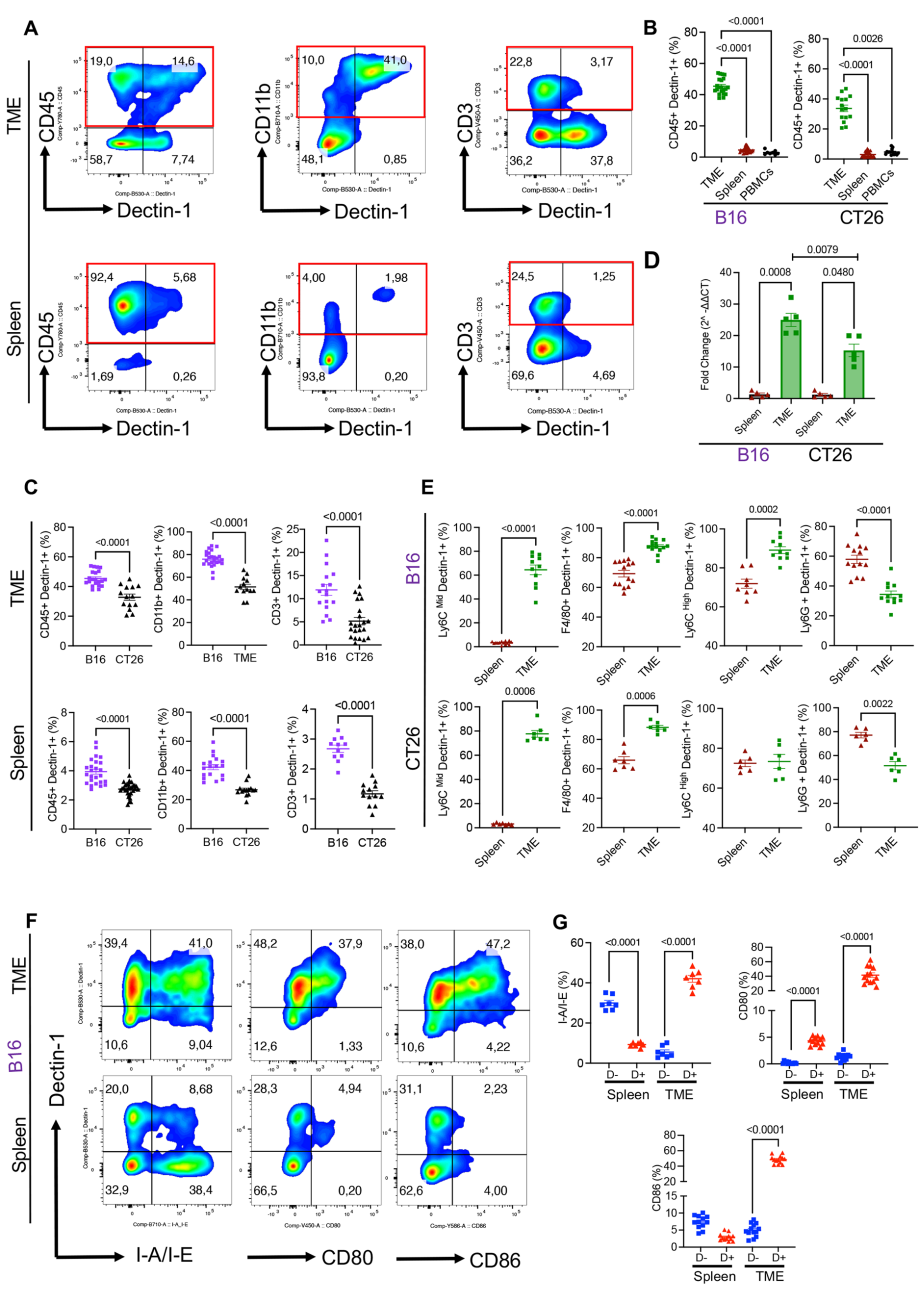
Dectin-1 is highly expressed on immune cells in the tumor microenvironment (TME). **A** Representative flow cytometry plots of the frequency of tumoral and splenic Dectin-1 expressing cells among CD45^+^, CD11b^+^, and CD3^+^ T cells. **B** Cumulative data of percentages of CD45^+^Dectin-1^+^ in the TME, spleen and peripheral blood of B16 and CT26 tumor-bearing mice. **C** Comparing the cumulative percentages of CD45^+^Dectin-1^+^, CD11b^+^Dectin-1^+^ and CD3^+^Dectin-1^+^ immune cells between CT26 and B16 tumor models. **D** Cumulative data of fold change in mRNA expression for clec-7 in the TME and spleen of B16 and CT26 tumor models. **E** Cumulative data of percentages of Dectin-1^+^ monocytes (Ly6C^Mid^), Dectin-1^+^ macrophages (F4/80^+^), Dectin-1^+^ M-MDSCs (Lyc6^High^) and Dectin-1^+^ G-MDSCs (LyG6^+^) subsets in the TME and spleen of CT26 and B16 tumor-bearing mice. **F** Representative flow cytometry plots for the co-expression of Dectin-1 with I-A/I-E, CD80 and CD86 in CD11b^+^ myeloid cells in the spleen and TME of B16 tumor-bearing mice. **G** Cumulative data of percentages of co-expression of Dectin-1^+^/Dectin-1- with I-A/I-E, CD80 and CD86 in CD11b^+^ myeloid cells in the spleen and TME of B16 tumor-bearing mice. *P* values were calculated using two tailed, Mann-Whitney *t* test (**C**, **E**). One-way ANOVA (**B**, **D**, **G**). Each dot represents an animal, and our data are obtained from multiple independent experiments, with at least 2 to 3 independent experiments conducted for each analysis. Representative plots are generated from the B16 model

The role of Dectin-1 in TME has been the subject of controversy. A few groups have reported a protective role for Dectin-1 against tumors [25, 26, 41] while others have shown that Dectin-1 aggravates tumour growth by switching M1 macrophages to M2 phenotype [17, 42]. To better understand the role of Dectin-1 in tumor immunogenicity, we investigated the frequency of Dectin-1^+^ immune cells in the tumour tissue, spleen, and peripheral blood of B16 melanoma and CT26 colorectal tumour models. Our analysis revealed that the TME in both tumour models had significantly higher Dectin-1^+^CD45^+^ immune cells than the spleen and peripheral blood (Fig. 1A, B, and Supplementary Fig. S1A, B). In particular, we found that CD11b^+^ cells were the most prominent cells expressing Dectin-1, and also a small portion of CD3^+^ T cells expressed Dectin-1 in the TME (Fig. 1A). Comparing two tumour models, we found that B16 melanoma-bearing mice had a remarkably larger proportion of Dectin-1 expressing cells among their total CD45^+^, CD11b^+^, and CD3^+^ cells in their spleens and tumour tissues compared to the CT26 colorectal tumour model (Fig. 1C). To confirm whether the intensity of Dectin-1 expression was different in immune cells, we also compared the mean fluorescence intensity (MFI) of Dectin-1 among CD45^+^ immune cells. Similarly, we observed that the intensity of Dectin-1 expression was greater in the B16 melanoma model than the CT26 colorectal cancer in the spleen, peripheral blood, and TME (Supplementary Fig. S1C). To examine the origin of Dectin-1^+^ myeloid cells, we measured the expression of Dectin-1 in splenocytes and peripheral blood mononuclear cells (PBMCs). This analysis revealed a significantly lower expression of Dectin-1 in total splenocytes and PBMCs compared to the TME resident cells in both tumor models (Supplementary Fig. S1D). These observations imply that the upregulation of Dectin-1^+^ expression might be a product of the TME. Parallel to the surface protein level, the expression of Dectin-1 at the gene level (celec7a gene) was significantly higher in sorted CD45^+^ immune cells from tumour tissues compared to their counterparts in the spleen (Fig. 1D). In agreement with protein levels, celec7a gene expression was significantly higher in sorted CD45^+^cells from the TME of B16 versus CT26 (Fig. 1D). To determine whether the frequency of Dectin-1 expressing cells influences the proportion of T cells in the TME, we analyzed the correlation between CD45^+^Dectin-1^+^ cells with total CD3^+^, CD4^+^ or CD8^+^ T cells in the B16 model that had significantly higher CD45^+^Dectin-1^+^ cells. We found a significant negative correlation between the frequency of CD45^+^Dectin-1^+^ with total CD3^+^ and CD8^+^ T cells but not CD4^+^ T cells in the B16 model (Supplementary Fig. S1E). In contrast, we observed a significant but positive correlation between the frequency of CD45^+^Dectin-1^+^ cells and CD3^+^ and CD8^+^ T cells but not CD4^+^ T cells in the CT26 tumour model (Supplementary Fig. S1F). This may suggest different roles for Dectin-1 in an immunogenic versus non-immunogenic model as reported elsewhere [17, 42]. Next, we quantified the expression of Dectin-1 in CD45^-^ cells in the TME that are supposedly tumor cells. We found that the majority of these cells expressed Dectin-1, however, CD45^-^Dectin^-^1 expressing cells were more abundant in B16 than CT26 model (Supplementary Fig. S1G). Finally, we assessed the expression of different co-inhibitory receptors/ligands in tumor cells, which revealed that these CD45^-^ cells predominantly expressed PD-L1 but not PD-L2, VISTA and Gal-9 (Supplementary Fig. S1H, I). Collectively, these results indicate a higher percentage of Dectin-1 expressing cells in the TME, particularly, the non-immunogenic TME (B16) comprised of more Dectin-1^+^ immune cells in both the TME and spleen than CT26.

### Tumour-associated myeloid cells express different levels of Dectin-1 in the TME

Unlike human myeloid cells, all subsets of myeloid cells in mice express different levels of Dectin-1 [43]. Therefore, we investigated the proportion of Dectin-1 expressing cells among different myeloid cells. We first characterized myeloid subsets including monocytes (CD11b^+^F4/80^-^ Ly6C^low/mid^Ly6G^-^), macrophages (CD11b^+^F4/80^+^), M-MDSCs (CD11b^+^F4/80^-^Lyc6^High^Ly6G^-^) and G-MDSCs (CD11b^+^F4/80^-^LyG6^+^Ly6C^-^) in the TME and spleen of both tumour models (Supplementary Fig. S2A). We found that in the B16 model, the proportion of monocytes was significantly lower in the TME compared to their counterparts in the spleen (Supplementary Fig. S2B). However, the proportion of M-MDSCs and G-MDSCs were significantly higher in the TME compared to their siblings in the spleen without any changes in the proportion of macrophages (Supplementary Fig. S2B). Almost the same pattern was observed in the CT26 model except the proportion of macrophages was significantly higher in the TME compared to the spleen (Supplementary Fig. S2B). Next, we evaluated the frequency of Dectin-1 expressing cells among different myeloid subsets. We observed significantly higher frequency of Dectin-1 expressing monocytes, TAMs, and M-MDSCs in tumor tissues of B16 mice compared to their siblings in the spleen (Fig. 1E and Supplementary Fig. S2C). Although the same pattern was noted for monocytes and macrophages in the CT26 model, the M-MDSC population didn’t show any difference regarding the frequency of Dectin-1 expression cells between TME and spleen (Fig. 1E). Interestingly, unlike M-MDSCs, the G-MDSC subpopulation had significantly lower proportion of Dectin-1 expressing cells in the TME compared to the spleen in both animal models (Fig. 1E). Next, we compared the percentages of Dectin-1^+^ myeloid subsets in two tumour models, which showed only a significant increase in the frequency of Dectin-1^+^ G-MDSCs in the CT26 mouse model (both in the TME and spleen) without any difference for other myeloid subsets (Supplementary Fig. S3A, B). Since it has been reported that TAMs also express high levels of CD206, a pathogen recognizing CLR, in cancer [44], we measured the frequency of CD206 expressing myeloid cells in the TME of the melanoma-bearing mice. However, we did detect a small portion of myeloid cells expressing this CLR or co-expressing it with Dectin-1 in tumor tissues (Supplementary Fig. S3C). Taken together, these findings support the abundance of Dectin-1 expressing myeloid cells in tumour tissues.

### Dectin-1^+^ myeloid cells are highly active and functional in the TME

**Fig. 2:**
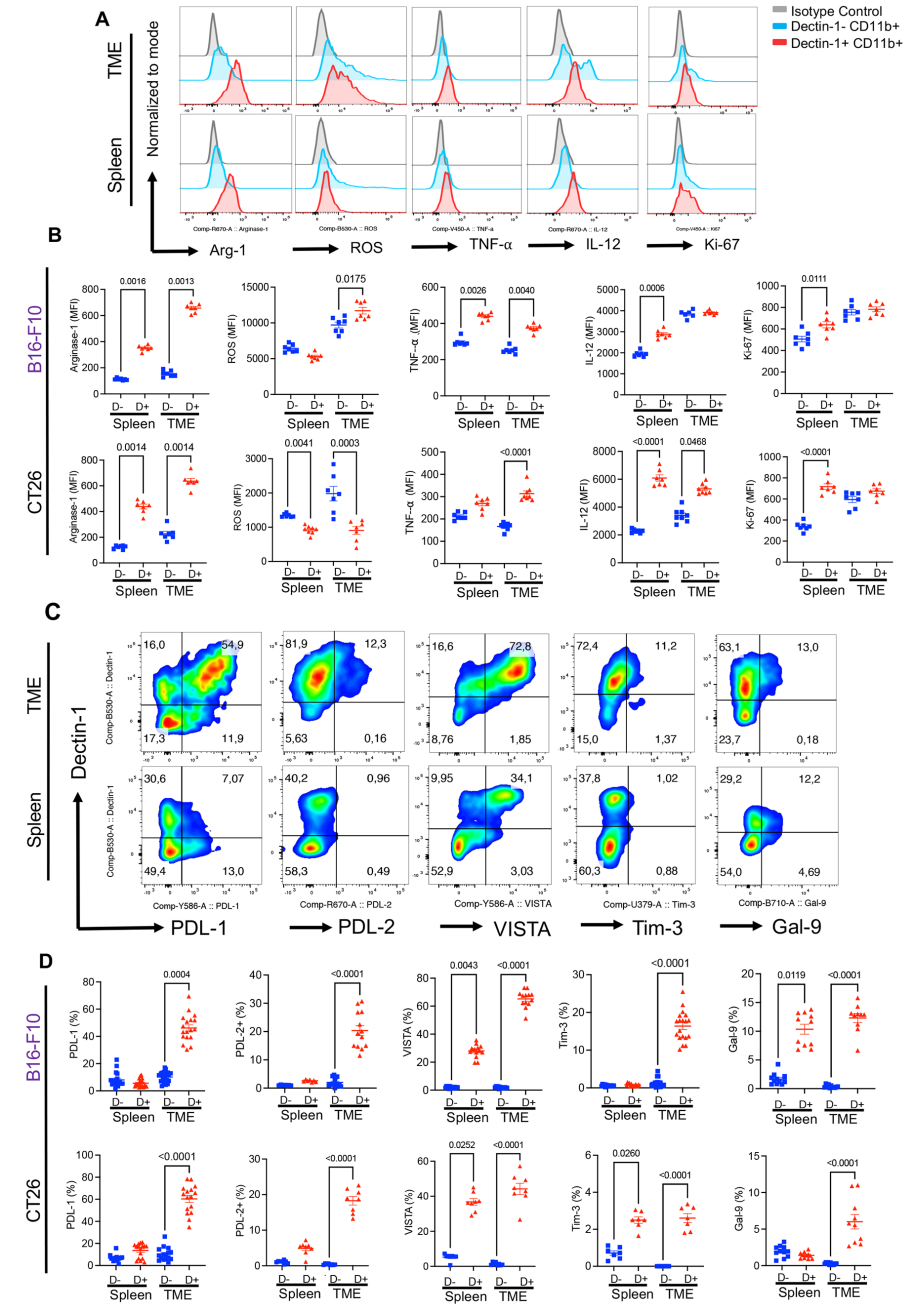
Dectin-1^+^ myeloid cells are highly active and functional in the TME. **A** Representative histogram plots of Arginase-1 (Arg-1), Reactive Oxygen Species (ROS), TNF-⍺, IL-12 and Ki-67 expression among Dectin-1^+^CD11b^+^ and Dectin-1-CD11b^+^ fractions of myeloid cells in the spleen and TME of B16 tumor model. **B** Cumulative data showing mean fluorescence intensity (MFI) of Arg-1, ROS, TNF-⍺, IL-12 and Ki-67 among tumoral and splenic Dectin-1^+^CD11b^+^ and Dectin-1-CD11b^+^ subsets in B16 and CT26 tumor models. **C** Representative flow cytometry plots for tumoral and splenic co-expression of Dectin-1 with PDL-1, PDL-2, VISTA, TIM-3 and Gal-9 among CD11b^+^ myeloid cells in the TME and spleen of B16 tumor model. **D** Cumulative data showing the percentages of PDL-1, PDL-2, VISTA, TIM-3 and Gal-9 expressing cells among Dectin-1^+^CD11b^+^ and Dectin-1-CD11b^+^ subsets in the spleen and TME of B16 tumor model. *P* values were calculated using One-way ANOVA (**B**, **D**). Each dot represents an animal, and our data are obtained from multiple independent experiments, with at least 2 to 3 independent experiments conducted for each analysis. Representative plots are generated from the B16 model

We aimed to characterize the immune profile of Dectin-1^+^ myeloid cells compare to their Dectin-1^-^ counterparts in the TME/spleen by measuring the expression levels of activation markers. Hence, we dichotomized total myeloid cells into Dectin-1^-^ and Dectin-1^+^ subsets and measured the expression levels of I-A/I-E, CD80, and CD86 among these subsets. Compared to splenic myeloid cells, we observed remarkable co-expression of I-A/I-E, CD80, and CD86 with Dectin-1 in tumoral myeloid cells in both tumour models (Fig. 1F, G and Supplementary Fig. S3D). However, this pattern was completely different in the spleen. For example, we observed significantly a lower percentage of I-A/I-E expressing cells, a higher frequency of CD80 expressing cells without any change in the proportion of CD86 expressing cells among Dectin-1^+^ myeloid cells in the spleen of B16 tumour model (Fig. 1F, G). However, Dectin-1^+^ splenic myeloid cells were significantly enriched with CD80 and CD86 expressing cells without any difference in the frequency of I-A/I-E expressing cells among Dectin-1^±^ cells in the CT26 model (Supplementary Fig. S3D). We further compared these activation markers among Dectin-1^+^ and Dectin-1^-^ myeloid cells in the immunogenic and non-immunogenic tumour models. We noticed a significant increase only in the frequency of I-A/I-E between Dectin-1^+^ and Dectin-1- myeloid cells in the TME of CT26 but the frequency of CD80 and CD86 expressing myeloid cells remained the same in both models (Supplementary Fig. S3E). Surprisingly, splenic Dectin-1^+^ myeloid cells in the CT26 model had significantly higher proportion of I-A/I-E, CD80, and CD86 expressing cells compared to their counterparts in the B16 model (Supplementary Fig. S3E). Furthermore, we measured the expression of Arginase-I (Arg-1), ROS, TNF-α, IL-12, IL-10, and Ki67 in freshly isolated cells without further stimulation *ex vivo*. In the B16 model, we observed that Dectin-1^+^ myeloid cells expressed significantly more Arg-I, ROS and TNF-α in the TME, while their counterparts in the spleen expressed significantly higher levels of Arg-I, TNF-α, IL-12 and Ki67 but not ROS (Fig. 2A, B). In the CT26 model, we observed that Dectin-1^+^ myeloid cells expressed significantly higher levels of Arg-I, TNF-α, IL-12 but lower ROS compared to their Dectin-1^-^ counterparts in the TME (Fig. 2B). However, Dectin-1^+^ myeloid cells in the spleen had significantly higher expression of Arg-I, IL-12 and Ki67 but lower ROS. At the same time the expression of Ki67 in the TME and TNF-α in the spleen remained unchanged (Fig. 2B). Next, we decided to compare Dectin-1^+^ and Dectin-1^-^ myeloid cells in terms of Arg-I, ROS, TNF-α, IL-12, and Ki67 expression in B16 versus CT26. These analyses revealed that myeloid cells regardless of Dectin-1 expression overall had elevated levels of ROS, TNF-α, IL-10, and Ki67 but lower IL-12 in both tissues (spleen and TME) in B16 than CT26 (Supplementary Fig. S4). Taken together, these results suggest that Dectin-1^+^ myeloid cells appear to exhibit an activated phenotype in the TME but they mainly appear to have immunosuppressive properties in the B16 tumour model, while in CT26 tumour model, they exhibit a pro-inflammatory phenotype. Therefore, Dectin-1 expressing myeloid cells might possess different biological properties in immunogenic and non-immunogenic tumor models.

### Dectin-1^+^ myeloid cells express substantial levels of different co-inhibitory receptors in the TME

**Fig. 3:**
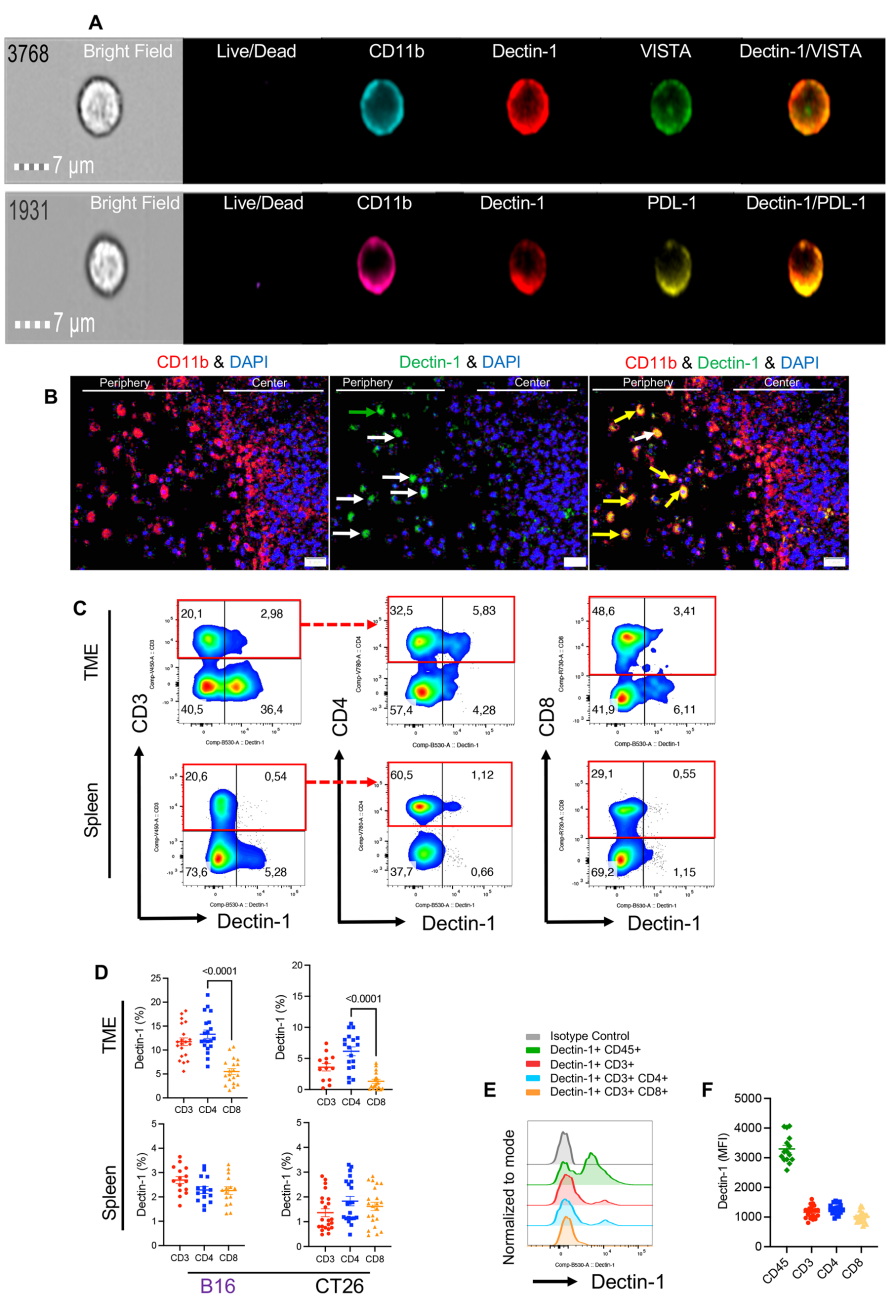
Dectin-1 is co-expressed/co-localized with VISTA and PDL-1^+^ on myeloid cells and a subset of T cells express Dectin-1 in the TME. **A** Image stream plots of surface co-localization of Dectin-1 and VISTA or Dectin-1 and PDL-1 on the surface of CD11b^+^ myeloid cells from the TME of B16 tumor model. **B** Representative images of the immunofluorescent staining (IF) for CD11b, Dectin-1 and DAPI in tumor tissue. **C** Representative flow cytometry plots of Dectin-1 expressing cells among CD3^+^, CD4^+^ and CD8^+^ T cells in the TME of B16 tumor model. **D** Cumulative data of percentages of Dectin-1^+^ cells among CD3^+^, CD4^+^ and CD8^+^ T cells in the TME and spleen of B16 and CT26 tumor models. **E** Representative histogram plots, and **F** cumulative data of MFI for Dectin-1 expression among CD45^+^ immune cells versus CD3^+^, CD4^+^ and CD8^+^ T cells in the TME of B16 tumor model. *P* values were calculated using One-way ANOVA (**D**). Each dot represents an animal, and our data are obtained from multiple independent experiments, with at least 3 independent experiments conducted for each analysis. Representative plots are generated from the B16 model

To further characterize immunological properties of Dectin-1^+^ myeloid cells in the TME, we subjected them to extensive analysis for the expression of different co-inhibitory molecules. We found that Dectin-1 was significantly co-expressed with PD-L1, PD-L2, VISTA, TIM-3, and Gal-9 in myeloid cells in the TME of both tumor models. However, this was not the case for Dectin-1 expressing myeloid cells in the spleen (Fig. 2C, D). In B16 model, we observed significant abundance of VISTA and Gal-9 expressing cells but in CT26, VISTA and TIM-3 expressing cells were significantly higher in Dectin-1^+^ myeloid cells in the spleen (Fig. 2C, D). Comparing the immunogenic and non-immunogenic tumor models, we found that Dectin-1^+^ myeloid cells were significantly enriched with VISTA, TIM-3 and Gal-9 expressing cells in the B16 model while PD-L1 expressing cells were more prominent in the CT26 model (Supplementary Fig. S5A). Next, we examined Dectin-1^+^ myeloid cells in terms of the expression of metabolic associated molecules such as CD39, CD26 and CD73 endonucleases in the TME. Although tumoral Dectin-1^+^ myeloid cells expressed elevated levels of CD39 and CD26 and CD73 (Supplementary Fig. S5B, C), their siblings in the spleen exhibited unchanged CD39, lower CD26, and higher CD73 expression (Supplementary Fig. S5C). The strong co-expression of Dectin-1 with PD-L1 and VISTA prompted us to evaluate possible co-localization of these molecules in myeloid subsets. These studies supported a substantial co-localization of Dectin-1 with both VISTA and PD-L1 in fresh myeloid cells from the TME (Fig. 3A, and Supplementary Fig. S5D). Taken together, our results imply a cross-talk between Dectin-1 and different co-inhibitory receptors/ligands, which may influence myeloid cell effector functions in the TME.

### Dectin-1^+^ myeloid cells are localized in the margin of the tumour tissue

The pattern of TILs is an important factor associated with the cancer progression [45, 46]. Likewise, immune disposition in the centre or periphery of the tumor, and the density and pattern of TILs are associated with the tumor outcomes [47, 48]. Therefore, we performed IF staining to determine the localization of Dectin-1^+^ myeloid cells (CD11b^+^) versus Dectin-1^-^ myeloid cells. We observed that although CD11b^+^ cells were distributed in the periphery and centre of the tumor, Dectin-1^+^CD11b^+^ cells were mainly deposited in the periphery (Fig. 3B). These observations indicate that Dectin-1^+^CD11b^+^ cells are located at the invasive tumor site (Supplementary Fig. S5E) rather than the centre in the B16 model.

### Dectin-1^+^ effector T cells are present in the TME and express co-inhibitory receptors

**Fig. 4:**
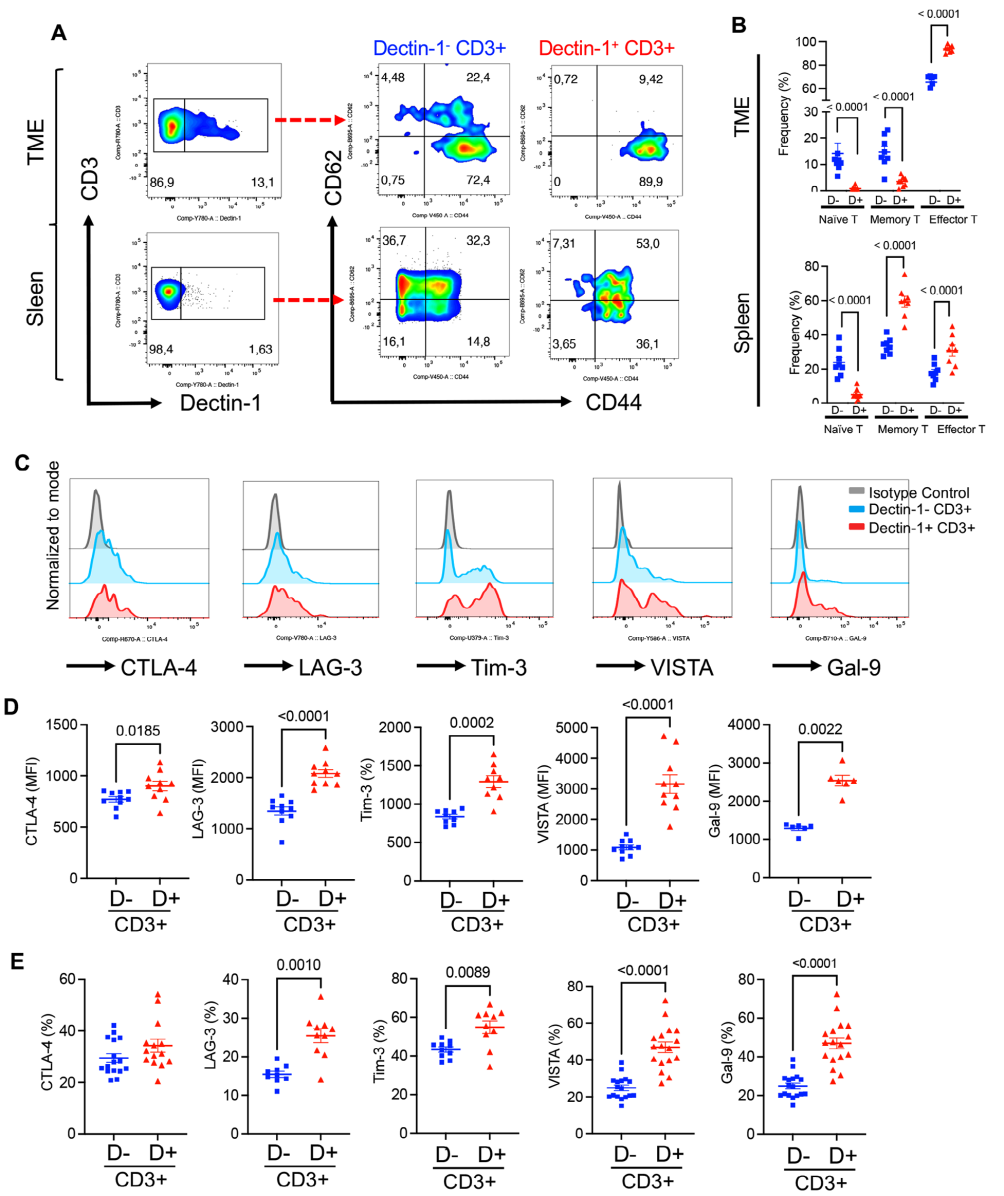
Dectin-1^+^ effector T cells are present in the TME and express co-inhibitory receptors. **A** Representative flow cytometry plots, and **B** cumulative data of percentages of Dectin-1^+^ naïve, memory and effector T cells in the TME and spleen of B16 tumor model. **C** Representative histogram plots, and **D** cumulative data of the MFI for CTLA-4, LAG-3, TIM-3, VISTA and Gal-9 in Dectin-1^+^CD3^+^ T cells versus Dectin-1-CD3^+^ T cells in the TME of B16 tumor model. **E** Cumulative data of percentages of CTLA-4, LAG-3, TIM-3, VISTA and Gal-9 expressing T cells among Dectin-1 + CD3^+^ T cells and Dectin-1-CD3^+^ T cells in the TME of B16 tumor model. *P* values were calculated using two tailed, Mann-Whitney *t* test (**D**, **E**). One-way ANOVA (**B**). Each dot represents an animal, and our data are obtained from multiple independent experiments, with at least 3 independent experiments conducted for each analysis. Representative plots are generated from the B16 model

It is reported that CCR6^+^IL-17-producing gamma-delta T cells express TLR-2 and Dectin-1 [49]. Based on this report, we decided to analyze the presence of Dectin-1^+^ T cells in B16 because of a greater frequency of these cells in this model (Fig. 1C). Our studies revealed the presence of a subset of CD3^+^ and subsequently CD4^+^ and CD8^+^ T cells expressing Dectin-1 in the TME, however, Dectin-1^+^ T cells were very scarce in the spleen (Fig. 3C, D). Although a subset of both CD4^+^ and CD8^+^ T cells in the TME expressed Dectin-1, CD4^+^ T cells were the dominant T cell subset having significantly higher frequency of Dectin-1^+^ cells in the TME of both B16 and CT26 models (Fig. 3C, D). However, the intensity of Dectin-1 was almost at the same level in CD3^+^, CD4^+^ and CD8^+^ T cells (Fig. 3E, F). Moreover, we compared the frequency of Dectin-1^+^ T cells in the TME and spleen of B16 versus CT26 tumour model. We found that Dectin-1^+^ CD3^+^, CD4^+^ and CD8^+^ were significantly enriched in the TME and spleen of B16 mice compared to CT26 (Supplementary Fig. S5F). Our further analysis revealed that Dectin-1^+^ T cells had a heterogeneous phenotype exhibited by the expression of different T cells-associated transcriptional factors such as GATA3, T-bet and RORγt (Supplementary Fig. S6A, B). These observations suggest that Dectin-1^+^ CD3^+^ T cells do not possess a unique transcriptional signature. Moreover, to better understand effector functions of Dectin-1^+^ CD3^+^ T cells, we subjected them to immune phenotyping and found that Dectin-1^+^ T cells had mainly an effector phenotype compared to Dectin-1^-^ CD3^+^ T cells in the TME (Fig. 4A, B). However, Dectin-1^+^ CD3^+^ T cells had a combination of central memory and effector phenotype in the spleen (Fig. 4A, B). Because of the effector phenotype of Dectin-1^+^ CD3^+^ T cells in the TME, we decided to subject them to further analysis for the expression of co-inhibitory receptors. We found that Dectin-1^+^ CD3^+^ T cells expressed significantly higher levels of CTLA-4, LAG-3, TIM-3, VISTA, and Gal-9 compared to their negative counterparts in the TME (Fig. 4C, D). Not only the intensity of co-inhibitory receptors expression but also the proportion of T cells expressing co-inhibitory receptors was significantly higher in Dectin-1^+^ T cells compared to their Dectin-1^-^ counterparts (Fig. 4E and Supplementary Fig. S6C). We observed almost the same pattern for the expression of these co-inhibitory receptors on CD4^+^ and CD8^+^ T cells in the TME as they exhibited significantly higher intensity of CTLA-4, LAG-3, TIM-3, VISTA and Gal-9 compared to Dectin-1^-^ CD4^+^ and CD8^+^ T cells in the TME (Supplementary Fig. S6D, E). Collectively these results reveal that Dectin-1^+^ T cells express elevated levels of co-inhibitory receptors compared to their Dectin-1^-^ siblings.

### The combination of curdlan and/or the anti-VISTA antibody enhances overall immune responses in the B16 model

**Fig. 5:**
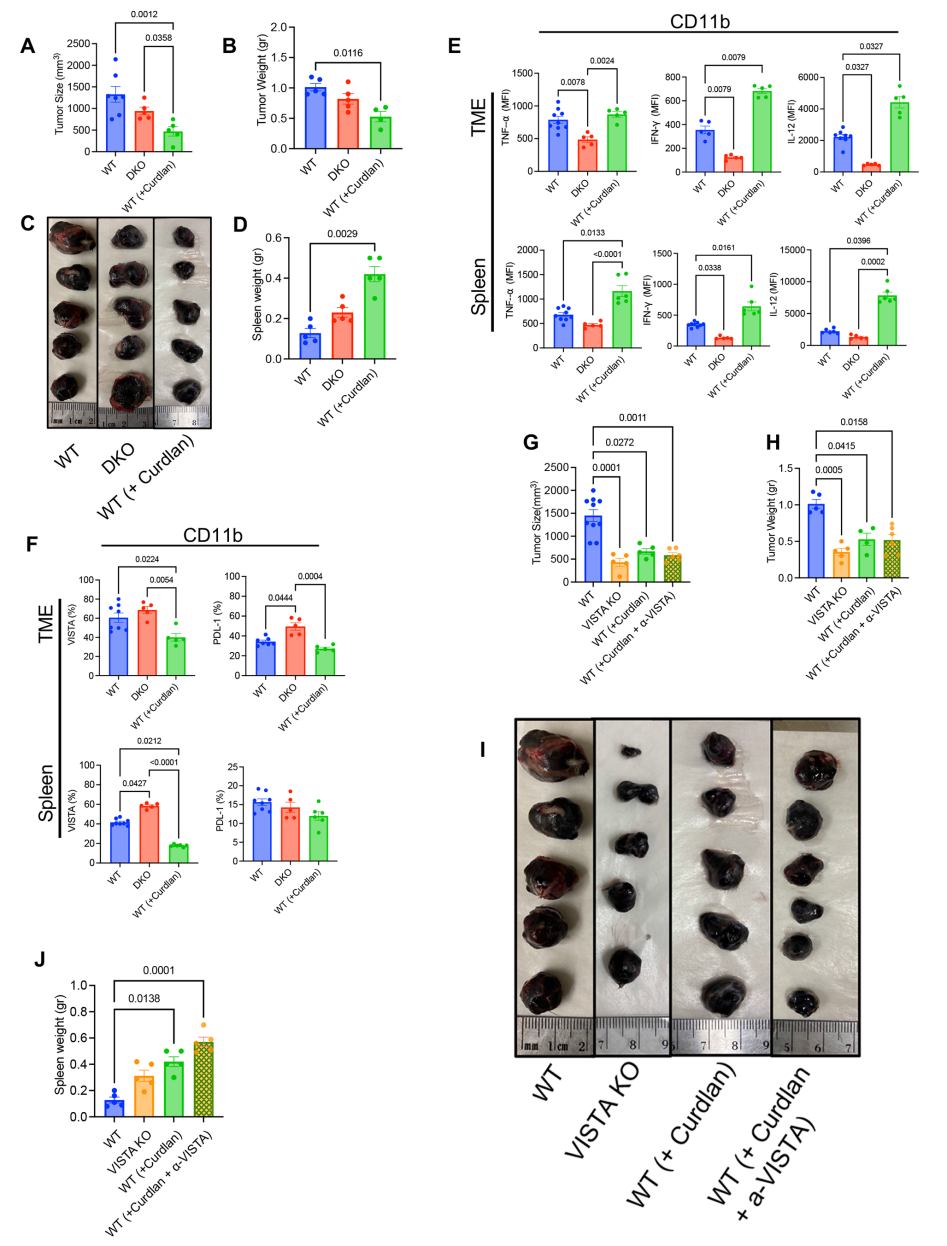
Targeting Dectin-1 by curdlan or the anti-VISTA antibody enhances overall immune responses in the B16 model. **A** Cumulative data showing tumor size in wild type (WT) tumor-bearing mice, Dectin-1 KO (DKO) tumor-bearing mice, and curdlan-treated tumor-bearing mice. **B** Cumulative data showing tumor weight in WT tumor-bearing mice, DKO tumor-bearing mice, and curdlan-treated tumor-bearing mice. **C** Representative pictures of dissected tumor tissues from WT tumor-bearing mice, DKO tumor-bearing mice, and curdlan-treated tumor-bearing mice. **D** Cumulative data showing the spleen weight in WT tumor-bearing mice, DKO tumor-bearing mice, and curdlan-treated tumor-bearing mice. **E** Cumulative data of the MFI for TNF-⍺, IFN-γ and IL-12 expression among CD11b + myeloid cells from the TME and spleen of WT tumor-bearing mice, DKO tumor-bearing mice, and curdlan-treated tumor-bearing mice. **F** Cumulative data of percentages of VISTA and PDL-1 expressing cells among CD11b^+^ myeloid cells from the TME and spleen of WT tumor-bearing mice, DKO tumor-bearing mice, and WT curdlan-treated tumor-bearing mice. **G** Cumulative data showing tumor size in WT tumor-bearing mice, VISTA KO tumor-bearing mice, WT curdlan-treated tumor-bearing mice and combined (curdlan + anti-VISTA antibody) treated WT tumor-bearing mice. **H** Cumulative data of tumor weight in WT tumor-bearing mice, VISTA KO tumor-bearing mice, WT curdlan-treated tumor-bearing mice and combined treated WT tumor-bearing mice. **I** Representative picture of dissected tumor tissue from WT tumor-bearing mice, VISTA KO tumor-bearing mice, WT curdlan-treated tumor-bearing mice and combined (curdlan ^+^ anti-VISTA antibody) treated WT tumor-bearing mice. **J** Cumulative data of spleen weight in WT tumor-bearing mice, VISTA KO tumor-bearing mice, WT curdlan-treated tumor-bearing mice and combined (curdlan ^+^ anti-VISTA antibody) treated WT tumor-bearing mice. *P* values were calculated using One-way ANOVA (**A**, **B**, **D**–**H**, **J**). Each dot represents an animal, and our data are obtained from multiple independent experiments. These data are representative of 3 independent experiments

Given the controversial role of Dectin-1 in cancer progression [17, 25], we decided to target Dectin-1 by curdlan, a commonly used agonist, in vivo. For this purpose, B16 tumor bearing wild type (WT) mice were treated (i.p.) with curdlan (15 mg/kg) starting one day after tumour inoculation every 3 days for a total of five treatments as illustrated in Supplementary Fig. S7A. Also, we used DKO mice as another control group to compare the absence versus antagozing of Dectin-1 in B16 model. We observed that the tumor volume was not significantly different in WT versus DKO mice, however, treatment with curdlan significantly reduced the tumor size and tumor weight in WT mice (Fig. 5A–C). In parallel, we found that curdlan-treated mice showed significantly enlarged spleen size, while no significant difference was noted between WT and DKO mice (Fig. 5D and Supplementary Fig. S7B). Further assessment of splenocytes and immune cells from the TME indicated that curdlan treatment significantly increased the proportion of CD4^+^ and CD8^+^ T cells in the TME, however, it did not impact the proportion of CD11b^+^ (Supplementary Fig. S7C). Also, we found that curdlan treatment significantly increased the proportion of CD4^+^ T cells and CD11b^+^ cells but in contrast reduced the frequency of CD8^+^ T cells in the spleen of mice (Supplementary Fig. S7C). Although the frequency of CD11b^+^ cells remained unchanged, this treatment was associated with increased TNF-α, IFN-γ and IL-12 expression in total CD11b^+^ cells in the TME and spleen of WT mice (Fig. 5E). We also noticed that curdlan treatment significantly reduced the percentages of VISTA^+^ and PD-L1^+^ CD11b cells in the TME but only VISTA^+^ CD11b^+^ cells in the spleen (Fig. 5F). It is worth mentioning that CD11b^+^ cells in DKO mice exhibited significantly lower expression of TNF-α, IFN-γ and IL-12 cytokines in their spleens and TMEs (Fig. 5E). We further analyzed the effects of curdlan treatment on T cell functions in tumor-bearing mice. We observed that expanded CD4^+^ T cells in curdlan treated mice expressed significantly higher levels of TNF-α (Supplementary Fig. S7D). However, this treatment had no significant effects on the expression of cytotoxic molecules (e.g. granzyme B and perforin) in CD8^+^ T cells in the TME and spleen (Supplementary Fig. S7E). Because of a substantial co-expression of VISTA with Dectin-1 on CD11b^+^ cells (Fig. 2C), and substantial reduction in its expression following curdlan treatment (Fig. 5F), we decided to better delineate the role of VISTA in this model.

**Fig. 6:**
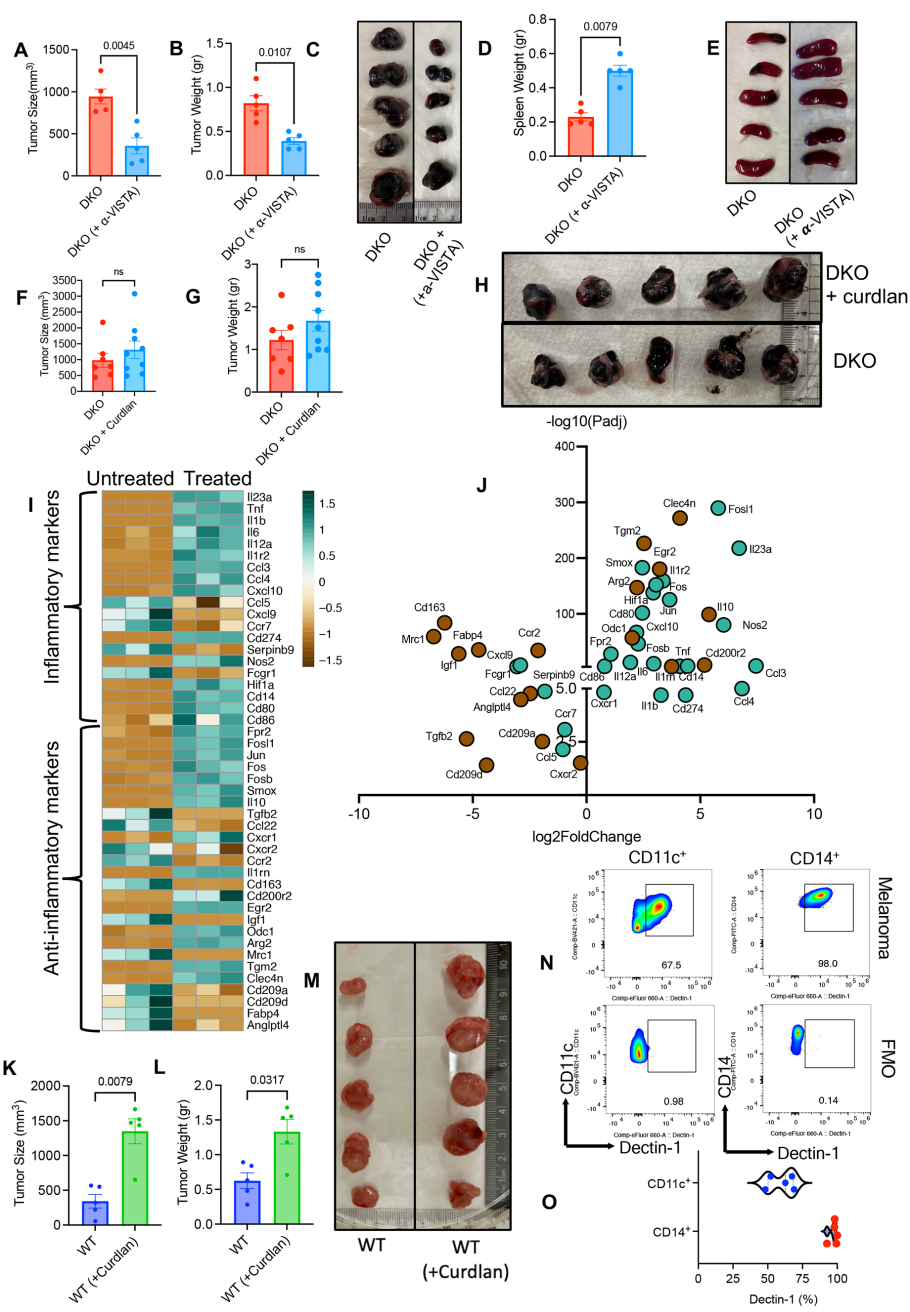
**A** Cumulative data of tumor size in DKO tumor-bearing mice vs DKO plus ⍺-VISTA antibody treated tumor-bearing mice. **B** Cumulative data of tumor weight in DKO tumor-bearing mice vs DKO plus ⍺-VISTA antibody treated tumor-bearing mice. **C** Representative pictures of dissected tumor tissues from DKO tumor-bearing mice vs DKO plus ⍺-VISTA antibody treated tumor-bearing mice. **D** Cumulative data of spleen weight in DKO tumor-bearing mice vs DKO plus ⍺-VISTA antibody treated tumor-bearing mice. **E** Representative pictures of dissected spleens from DKO tumor-bearing mice and DKO plus ⍺-VISTA antibody treated tumor-bearing mice. **F** Cumulative data of tumor size in DKO tumor-bearing mice (B16-F10) treated with and without curdlan. **G** Cumulative data of tumor weight in DKO tumor-bearing mice (B16-F10) treated with or without curdlan. **H** Representative pictures of dissected tumor tissues from DKO tumor-bearing mice treated with and without curdlan. **I** Heatmaps of genes associated with M1 and M2 macrophages in myeloid cells stimulated with or without curdlan in vitro. **J** Volcano plots of differentially expressed genes associated with M1 and M2 macrophages in myeloid cells stimulated or unstimulated with curdlan. **K** Cumulative data of tumor size, and **L** tumor weight in CT26 tumor-bearing mice treated (WT + curdlan) or untreated (WT). **M** Representative pictures of dissected tumors in CT26 tumor-bearing mice treated (WT + curdlan) or untreated (WT). **N** Representative flow cytometry plots, and **O** cumulative data for the expression of Dectin-1 in human CD14 + and CD11c + cells from melanoma patients. *P* values were calculated using two tailed, Mann-Whitney *t* test (**A**, **B**, **D**, **F**, **G**, **K**, **L**, **O**). Each dot represents an animal, and our data are obtained from multiple independent experiments. These data are representative of 3 independent experiments. Each dot represents a human study subject (**O**). Florescence minus one (FMO)

It is well-documented that blocking VISTA can result in a robust immune response against cancer [50, 51]. Therefore, we decided to perform a comprehensive study by investigating the role of curdlan in WT and VISTA KO mice. For this purpose, we first compared tumor size in WT treated with curdlan (15 mg/kg) (Supplementary Fig. S7A), VISTA KO mice, treated WT with curdlan (15 mg/kg) plus anti-VISTA antibody (250 µg/mouse) (Supplementary Fig. S8A). In agreement with our observations in previous experiments (Fig. 5A, B), we found a significant reduction in the tumor size and weight in WT mice treated with curdlan (Fig. 5G–I). As anticipated, the tumor size/weight was significantly smaller in VISTA KO mice compared to their WT counterparts (Fig. 5G–I). However, the combination therapy with the anti-VISTA neutralizing antibody plus curdlan did not induce any synergistic effects on reducing the tumor mass (Fig. 5G–I). We noted that a reduction in tumor volume was associated with an increase in the spleen size (Fig. 5J and Supplementary Fig. S8B). Analysis of immune cell proportions in the TME revealed significantly higher percentages of CD4^+^ and CD8^+^ T cells in the TME of VISTA KO and WT mice treated with curdlan (Supplementary Fig. S8C). At the same time, we noted an increase in the NK cell population in the TME of only VISTA KO and WT treated with curdlan plus anti-VISTA antibody (Supplementary Fig. S8C). Also, we found an increase in CD4^+^ T cell percentages in WT treated mice once treated with curdlan without any significant increase in CD8^+^ T cells in the spleen (Supplementary Fig. S8C). Similarly, we observed an increase in NK cell proportion in the TME of VISTA KO and WT mice treated with the anti-VISTA antibody plus curdlan compared to the WT group (Supplementary Fig. S8C). Although CD11b^+^ cells remained unchanged in the TME, we found a significant expansion of these cells in the spleen of WT mice treated with curdlan and those treated with curdlan plus anti-VISTA antibody compared to the WT group (Supplementary Fig. S8C). In terms of cytokine expression, we did not see a clear picture when different groups were compared to each other. Unlike curdlan-treated mice that showed enhanced IFN-γ and IL-12 expression in their CD11b^+^ in the TME and spleen, VISTA KO and double treated mice exhibited higher IFN-γ in CD11b^+^ in their spleens only (Supplementary Fig. S9A). In CD4^+^ T cells, when four groups were compared to each other in terms of cytokine expression we did not observe a clear picture; but we noted a higher expression of TNF-α and IFN-γ in VISTA KO compared to the WT group in the TME (Supplementary Fig. S9B). It appeared that CD8^+^ T cells exhibited a greater GzmB and perforin expression only in the VISTA KO group (Supplementary Fig. S9C). Since VISTA was highly co-expressed with Dectin-1 (Fig. 2C, D), we decided to investigate whether neutralizing VISTA can influence the tumor growth in DKO mice. We found that treatment of DKO with the anti-VISTA antibody (Supplementary Fig. S9D) resulted in a significant reduction in the tumor size/tumor weight (Fig. 6A–C) but increased in spleen weight (Fig. 6D, E) and frequency of CD8^+^ T cells (Supplementary Fig. S9E) without any change in the frequency of CD4^+^ T cells, NK cells and CD11b^+^ cells. Interestingly, we observed that CD8^+^ T cells in DKO mice treated with the anti-VISTA antibody expressed significantly higher levels of perforin and GzmB in the TME and spleen (Supplementary Fig. S9F). Moreover, we noted higher expression of Ki67, TNF-α and IFN-γ expression in CD11b^+^ cells of DKO mice once treated with the anti-VISTA antibody (Supplementary Fig. S10A). Finally, we treated VISTA KO mice with curdlan according to the timelines described in Supplementary Fig. S7A. However, curdlan treatment had no significant impact on the tumor size (Supplementary Fig. S10B, C) and spleen size in VISTA KO mice (Supplementary Fig. S10D, E). Although curdlan treatment significantly increased the proportion of CD11b^+^ cells in the TME and spleen, it was at the expense of a reduction in CD8^+^ T cells frequency (Supplementary Fig. S10F, G). Furthermore, we noted that CD8^+^ T cells in VKO (VISTA KO) treated mice with curdlan exhibited reduced perforin and GzmB expression (Supplementary Fig. S10H). Finally, in lights of the interaction of curdlan with TLR-4 and TLR-2 [52, 53], we treated DKO mice with curdlan as outlined in Supplementary Fig. S7a. Interestingly, we did not observe any significant difference in the tumor size/weight and spleen size in DKO mice (Fig. 6F–H and Supplementary Fig. 10I). These observations support the specificity of curdlan and Dectin-1 interaction in our model.

### Curdlan treatment increases the expression of genes associated with neutrophil activation

Next, we examined gene expression through RNAseq analysis in Bone-marrow derived neutrophils from wild type mice once treated with curdlan, using a publicly available dataset (GSE148850) [54]. We were able to detect the difference between treated and untreated cells. We observed that curdlan upregulated several genes associated with pro-inflammatory cytokines, such as IL-23a, TNF, IL-1-β, IL-6, IL-12a, and IL-1R2. Notably, TGF-β2, a hallmark of anti-inflammatory, was downregulated (Fig. 6F, G and Supplementary Fig. 10J). Similarly, the upregulation of several chemokines and chemokine ligands (e.g. CXCL10, CCL-3, and CCL4) [55] and the downregulation of CCL22 [56] support neutrophils activation following curdlan treatment (Fig. 6I, J and Supplementary Fig. 10J). Of note, curdlan treatment upregulated the expression of PD-L1 and PD-L2, which is in line with IFN-γ signaling [57] (Fig. 6I, J). Moreover, the upregulation of co-stimulatory molecules CD80, CD86, and CD14, but the downregulation of markers such as CD209, mannose receptor 1(MRC1, CD206), and CD163 suggest that neutrophils become more activated [58] upon treatment with curdlan (Fig. 6I, J). Likewise, the upregulation of Jun and Fos as main components of the AP-1 transcription factor complex and nitric oxide synthase (NOS) further support activation of neutrophils [59].

### Curdlan promotes pathways associated with immune activation and inflammatory responses

We performed pathways analysis using the Ingenuity Pathway Analysis (IPA, QIAGEN) software to determine altered pathways associated with curdlan stimulation of BM-derived neutrophils as we have reported elsewhere [36]. We found several key pathways indicate heightened immune activation and inflammatory responses, such as Toll-like receptor signaling, IL-6 signaling, TNF signaling, and pathways involved in cytokine storm signaling (Supplementary Fig. 11). These pathways typically trigger a cascade of events leading to increased production of pro-inflammatory cytokines, chemokines, and other mediators, resulting in the recruitment and activation of immune cells to the site of inflammation.

Additionally, we found significant upregulation of pathways involved in cellular stress responses, such as NFEL2L2 regulating antioxidant/detoxification enzymes and unfolded protein response, implies that neutrophils are responding to curdlan by activating protective mechanisms to mitigate cellular damage and maintain cellular homeostasis (Supplementary Fig. 11). Moreover, activation of pathways such as acute phase response signaling, immunogenic cell death signaling, unfolded protein response, and pyroptosis signaling suggests that neutrophils are responding to cellular stress and potential damage. This could involve mechanisms to repair cellular damage, trigger cell death, or eliminate damaged cells. Furthermore, these analyses revealed the upregulation of pathways like CGAS-STING, IL-33, and TREM1 signaling indicates activation of immune cell signaling pathways involved in detecting pathogens, activating immune responses, and modulating inflammation (Supplementary Fig. 11). Finally, we found upregulation of pathways such as amino acids regulate mTORC1 and iron uptake and transport suggest potential metabolic reprogramming in activated neutrophils to support increased energy demands and cellular functions associated with immune activation (Supplementary Fig. 11).

In contrast, the downregulation of pathways associated with anti-inflammatory mechanisms, such as IL-10 signaling and PPAR signaling, further supports the notion of a shift towards a more pro-inflammatory state in neutrophils following curdlan stimulation (Supplementary Fig. 11). Given the collective upregulation of pathways associated with immune activation, inflammation, cellular stress responses, and metabolic regulation it is reasonable to suggest that neutrophils become more activated in response to curdlan stimulation in vitro.

### Targeting Dectin-1 with curdlan resulted in tumor progression in the CT26 model

Considering that Dectin-1 is also expressed on different immune cells in TME of the CT26 model (Fig. 1B–D) and β-glucans are abundant in foods and food supplements such as mushrooms and herbal medicine, we investigated its role in this colorectal model. We were unable to examine the effect of Dectin-1 deficiency in the CT26 model because our DKO mice are on B57BL/6 background. However, we investigated the influence of Dectin-1 stimulation by curdlan using the same treatment regimens used in B16-F10 (Supplementary Fig. S7A). Surprisingly, we observed that stimulation of Dectin-1 resulted in a significant increase in tumor size/weight in this model (Fig. 6K–M). This is consistent with a recent report that indicates blocking Dectin-1 inhibits tumor progression in colorectal cancer [60].

### Myeloid cells in human PBMCs express Dectin-1

To determine whether myeloid cells in humans similar to mice express Dectin-1, we examined the expression of Dectin-1 in blood monocytes (CD14) and dendritic cells (CD11c). We found that almost the entire CD14+ subset and the majority of CD11c+ cells in patients with melanoma express Dectin-1 (Fig. 6N, O). However, the expression of Dectin-1 in human peripheral blood T cells was negligible.

## Discussion

Targeting the TME is increasingly considered as a promising approach for cancer immunotherapy [61]. Although using ICBs as mono or combination therapies (e.g. PD-1/PD-L1 and CTLA-4) have shown promising results in a few types of cancers, success requires a deeper understanding of the TME and factors that can be targeted to enhance antitumor immune responses. The innate immune cells including DCs, tissue-resident macrophages, and recruited monocytes play an essential role in enhancing or impairing anti-tumour immunity [62, 63]. Recent pieces of evidences suggest that targeting innate immune cells in the TME is an alternative approach for effective anti-tumour immunity [64–66]. In this study, we focused on Dectin-1^+^ expressing cells in the TME and periphery of CT26 and B16 tumour models. We also examined the potency of targeting Dectin-1 as a complementary target in the combined immunotherapy setting. We found that the least immunogenic tumour model, B16, had significantly higher proportion of Dectin-1 expressing myeloid cells in the TME than the CT26 tumour model, which is considered an immunogenic model [67]. Considering that the frequency of Dectin-1^+^ myeloid cells in the TME was negatively correlated with T cell infiltration suggest that higher abundance of Dectin-1 myeloid cells might be considered as a hallmark of non-immunogenic tumours. In agreement, Dectin-1 expression by clear cell renal cell carcinoma is reported to be associated with adverse postoperative prognosis [42]. Mechanistically the interaction of Dectin-1 with Gal-9 results in tolerogenic macrophage programming and the impairment of the adaptive immune system, which accelerates tumour progression in PDA model [17]. However, our observations may not support a crucial role for Gal-9 in B16 and CT26 tumor models because its expression in tumor cells was negligible and a small portion of myeloid cells in the TME and spleen expressed Gal-9. However, we found that myeloid cells in the TME but not spleen and peripheral blood profoundly expressed Dectin-1, which was more prominent at the protein and gene levels in the B16 than the CT26 model. Our results are consistent with another report showing that this molecule was predominantly expressed by different myeloid subsets, including macrophages, monocytes and neutrophils [43]. In particular, we noted a differential expression of Dectin-1 in myeloid subsets in the TME. This implies that Dectin-1 may exhibit different roles in myeloid subsets in the TME. For example, it is reported that yeast-derived Particulate β-Glucan via ligation with Dectin-1 induces apoptosis in G-MDSCs but promotes the maturation of M-MDSCs [68].

Dectin-1 is an activation receptor that can stimulate immune cell maturation and enhance cytokines and chemokines production [69]. As Dectin-1 was found predominantly in tumor cells in our study, tumor cells might get activated through this signal and influence the immunoregulatory network of the TME. Although we were unable to identify the mechanism(s) underlying the upregulation of Dectin-1 in myeloid and tumor cells, it is reported that macrophages treated with IL-4 and IL-13 upregulate the expression of Dectin-1 [70]. Therefore, the cytokine milieu of the TME might be a potential factor in the upregulation of Dectin-1 in immune and non-immune cells. The TME hosts a heterogeneous population of myeloid cells with different dynamics and plasticity. In our research, however, Dectin-1 expression was predominantly associated with an activated myeloid cell phenotype in the TME as characterized by the expression of CD80, CD86 and I-A/I-E. It is possible to speculate that overexpression of antigen presentation molecules (e.g. MHC-I and II) and co-stimulatory molecules (e.g. CD80 and CD86) can enhance anti-tumour immune response [71, 72] in the melanoma model. In agreement, our RNAseq analysis supported that the stimulation of Dectin-1 preferentially promotes an activated neutrophil phenotype. Further analyses revealed that curdlan enhances pro-inflammatory response, immune activation, and metabolic regulation as potential pathways associated with a protective mechanism in the B16 model. Although these observations are informative, such data are obtained from tumor-free mice. Therefore, further studies are required to investigate the effects of Dectin-1 stimulation in tumor-educated myeloid cells in different cancer models. In contrast, it appears that Dectin-1^+^ myeloid cells exhibit an anti-inflammatory profile in the CT26 tumour model, as evidenced by tumor progression upon curdlan treatment. It is worth mentioning that Arg-I and Arg-II hydrolyze L-arginine, a dibasic cationic amino acid, to L-ornithine and play an important immunosuppressive role in the TME [73]. In humans, arginine deprivation inhibits T cell proliferation through decreasing CD3ζ-chain expression and prevents the cycle regulators cyclinD3 and cdk4 [74–76]. Hence, a higher expression of Arg-I by Dectin-1 expressing suggests that these cells may exhibit an inhibitory signal and suppression of Arg-1 could amplify the adaptive immune response in the TME [77]. ROS is another immunomodulatory component of the TME, primary released by myeloid cells, which triggers genome-wide DNA mutation, genomic instability and epithelial mutagenesis [78–80]. ROS could act as a stimulatory or an inhibitory molecule depending on the cancer type and its concentration. By analysing the ROS expression in Dectin-1^+^ myeloid cells in CT26 and B16 tumour models, we found that myeloid cells in the latter model produced a significant amount of this molecule. It is possible to speculate that lower ROS expression in Dectin-1^+^ myeloid cells in CT26 may reduce ROS-mediated cell death in colon cancer [81]. In contrast, higher expression of ROS in the B16 model may enhance DNA damage [82–84]. Obviously, further in deep analysis will be required to better characterize the functional properties of cancer-induced Dectin-1^+^ myeloid cells in different cancer types.

Examination of the TME Dectin-1^+^ myeloid cells revealed that this subset extensively expresses PD-L1, VISTA, and moderately PD-L2, TIM-3, and Gal-9. The expression of co-inhibitory receptors is regarded as the hallmark of T cell exhaustion and blockade of immune checkpoints (e.g. PD-1/PD-L1 and CTLA-4) has achieved considerable success in the treatment of different types of solid cancers [85]. Hence, Dectin-1^+^ myeloid cells by possessing elevated levels of co-inhibitory receptors/ligands might exhibit a broader role in immune regulation in the TME. One of the most significant observations in this study was the discovery of Dectin-1/VISTA and Dectin-1/PD-L1 co-expression/co-localization in myeloid cells from the TME. These results indicate a novel mechanism beyond the Dectin-1 signaling in modulating T cell plasticity in the TME. For example, Dectin-1^+^ myeloid cells may via PD-L1/PD-L2 suppress PD-1 expressing T cells in the TME [85]. Similarly, Gal-9 ligation with TIM-3 can induce an inhibitory signal and has been considered to be an exhaustion mechanism for antigen-specific CD8^+^ T cells [86, 87]. Moreover, recent studies have revealed that Gal-9 interaction with PD-1 and Gal-9 expressing T cell exhibit an impaired phenotype [35, 88–90]. Of note, Gal-9 interacts with different receptors and its interaction experts an inhibitory effect to Dectin-1 signaling, which promotes pancreatic carcinoma [17]. Another important observation of this study was the discovery of profound Dectin-1 and VISTA co-expression in myeloid cells not only in the TME but also the spleen of tumor-bearing mice. Although the expression of VISTA in myeloid cells and regulatory T cells in the TME has been reported [91], co-expression of Dectin-1 and VISTA has never been documented. VISTA is unique among other immunoglobulin superfamily molecules but lacks classic ITIM or immunoreceptor tyrosine-based switch motifs (ITSM) [92, 93]. This may explain dual role for VISTA in myeloid cells or T cells as a ligand or receptor, respectively. The inhibitory function of VISTA has widely been studied in cancer models [94–96]. For instance, VISTA inhibits the activation of MAP kinases and NF-κB signalling cascades and blocking VISTA amplifies TLR/MyD88-mediated pathway in myeloid cells [97]. Given that VISTA and PD-L1 were highly co-expressed with Dectin-1 in myeloid cells of B16 model, we postulated that Dectin-1 may enhance tumor progression. Surprisingly, we found that DKO mice had impaired immune cell activation and infiltration into the TME. This is in contrast to a report showing a tolerogenic role for Dectin-1 signaling in PDA [17]. However, we found that stimulation of Dectin-1 by curdlan enhanced anti-tumor immunity and reduced tumor progression. Additional evidence supports our observations that Dectin-1 stimulation in myeloid cells is essential for NK-cell-mediated tumor cytotoxicity [25]. It is reported that Dectin-1 activation in this model results in the activation of the interferon regulatory factor 5 transcriptional factor and downstream gene associated with enhanced NK cell tumoricidal activity [25]. Also, Dectin-1 ligation with β-glucan has been reported to confer protection against lung and mammary tumors in mice [98]. Similarly, studies in human subjects support a protective role for Dectin-1 signaling against cancer [99, 100]. Interestingly, we found that Dectin-1 expressing myeloid cells were enriched in the periphery of the tumor. Therefore, targeting these cells might be more accessible from the therapeutic standpoint, which can overall enhance the anti-tumor immunity. Beyond discovering a protective and significant role for Dectin-1 signaling in myeloid cells in the TME, one of the most exciting findings in our study is the downregulation of PD-L1 and VISTA upon curdlan-induced myeloid cells activation. Thus, these data might have far-reaching implications that imply a wider role for targeting Dectin-1 in the TME. Although VISTAKO mice exhibited a robust anti-tumor immune response against tumor, which agrees with other reports [67, 92], ligation of Dectin-1 with curdlan did not influence the tumor growth in these mice. Therefore, further studies are required to test different treatment regiments to determine whether combining dietary β-glucan structures alongside anti-VISTA and/or anti-PDL-1 antibodies enhance anti-tumour immunity.

Moreover, we discovered a subset of Dectin-1 expressing T cells, mainly CD4^+^ T cells, in the TME. It is worth mentioning that these cells were more abundant in the TME of the B16 model. In contrast to a previous report [49], our results show that Dectin-1^+^ T cells are heterogeneous but express substantial levels of co-inhibitory receptors. Although upregulation of co-inhibitory receptors is the hallmark of T cell exhaustion, we found that Dectin-1^+^ T cells did not exhibit such phenotype. This might be due to the nature of our studies that terminated by 16 days. These observations suggest that these T cells could be targeted by curdlan similar to myeloid cells. However, further studies are needed to better characterize effector functions of Dectin-1^+^ T cells in other animal tumor models and human cancers.

Our findings highlight the complex network of immunosuppressive pathways present in the TME that is unlikely to be overcome with a single immunotherapy target. Therefore, in addition to ICBs, Dectin-1 would be an attractive target for future immunotherapy development. Stimulation of Dectin-1 may convert polarized M2 macrophages into an M1-phenotype [26] likely to have synergistic efficacy with ICBs. It is conceivable that Dectin-1^+^CD8^+^ T cells expressing co-inhibitory receptors eventually become exhausted in the context of chronic conditions. Nevertheless, tumor progression following the stimulation of Dectin-1 in the CT26 model illustrates the complexity of myeloid cells manipulations. Taken together, stimulating myeloid cells via curdlan not only enhances antigen presentation but also reprograms tumour-associate myeloid cells toward an inflammatory phenotype in the melanoma model. This is highly important for melanoma patients because acquired resistance to ICBs is associated with defects in antigen presentation [101]. The abundance of Dectin-1 expressing myeloid cells in the peripheral blood of melanoma patients reinforces this hypothesis. Therefore, targeting Dectin-1 (e.g. curdlan) can result in a robust innate and adaptive immune response against tumour cells. In agreement with our results, a study suggested that durable regression of melanoma tumors requires concurrent immunotherapy that engages both innate and adaptive immune responses [102].

However, there are several limitations in our study, one of which is single-centred treatment design. Also, because of the animal ethics requirements we were unable to keep tumor-bearing mice for a longer period of time to determine the role of targeting Dectin-1 in a more chronic condition. Moreover, we were unable to investigate the exact mechanism of Dectin-1 expressing myeloid and T cells in the TME, which merits further investigations. The colocalization of Dectin-1 with PD-L1 and VISTA requires further confirmatory studies. Finally, human studies were performed on a limited number of melanoma patients and we did not have access to tumor biopsies to examine the expression level of Dectin-1 in myeloid cells the TME. Such studies are needed to better comprehend the role of Dectin-1 in human tumor tissue.

## Electronic supplementary material

Below is the link to the electronic supplementary material.

Supplementary Material 1

Supplementary Material 2

## Data Availability

All data related to this study are included in the main and Supplementary figures. Bulk RNA sequencing was conducted using a publicly available dataset (GSE148850).
