# Supplementary material for: Targeting Dectin-1 and or VISTA enhances anti-tumor immunity in melanoma but not colorectal cancer model
Source: Cell Oncol (Dordr). 2024 Apr 26;47(5):1735–56. doi: 10.1007/s13402-024-00950-w (PMC11467025; doi:10.1007/s13402-024-00950-w)
Supplement: Supplementary file 1 — Supplementary Material 1 [file 13402_2024_950_MOESM1_ESM.pdf]

Supplemental Figure legends

Supplemental figure S1. (**A**) Representative flow cytometry plots showing the gating strategy for immune cells in the TME and spleen. (**B**) Representative flow cytometry plot and cumulative data showing Dectin-1 expression on CD45+ immune cells in the peripheral blood of B16 and CT26 tumor models. (**C**) Cumulative data comparing the MFI of Dectin-1 in B16 and CT26 tumor models in the TME, spleen and blood. (**D**) Cumulative data comparing the MFI of Dectin-1 on total immune cells (CD45+) between TME, spleen and blood of B16 and CT26 tumor models. (**E**) Cumulative data of correlations between the frequency of total CD45+Dectin-1+ immune cells and CD3+, CD8+, and CD4+ T cells in the B16 tumor model. (**F**) Cumulative data of correlations between the frequency of total CD45+Dectin-1+ immune cells and CD3+ T cells and CD8+ T cells in the CT26 tumor model. (**G**) Representative flow cytometry plot and cumulative data of percentage of Dectin-1+ cells among live CD45- cells in the TME of B16 and CT26 tumor models. (**H**) Representative flow cytometry plots of co-expression of Dectin-1 with PDL-1, PDL-2, VISTA and Gal-9 on CD45- live cells in the TME of the B16 tumor model. (**I**) Cumulative data showing percentages of PDL-1, PDL-2, VISTA and Gal-9 on live CD45- cells in the TME of B16 tumor model. *P* values were calculated using two tailed, Mann-Whitney *t* test (**C**). The Spearmen correlation test (**E,F**).

Supplemental figure S2. (**A**) Representative flow cytometry plots of the gating strategy for myeloid subsets in the spleen and TME. (**B**) Cumulative data showing frequency of monocyte, macrophages, M-MDSCs and G-MDSCs in the spleen and TME of B16 and CT26 tumor models. (**C**) Representative flow cytometry plots of percentages of Dectin-1 expressing monocytes, macrophages, M-MDSCs and G-MDSCs in the TME and spleen. *P* values were calculated using One-way ANOVA (**B**).

Supplemental figure S3. (**A**) Cumulative data comparing B16 and CT26 tumor models for percentages of Dectin-1 expressing monocytes, macrophages, M-MDSCs and G-MDSCs in the TME. (**B**) Cumulative data comparing B16 and CT26 tumor models for expression of Dectin-1 in monocyte, macrophages, M-MDSCs and G-MDSCs populations in spleen. (**C**) Representative flow cytometry plots and cumulative data for the expression of CD206 in CD11b+myeloid cells, and co-expression of Dectin-1 with CD206 on CD11b+ myeloid cells in the TME. (**D**) Cumulative data showing percentages of I-A/I-E, CD80 and CD86 expressing cells among Dectin-1+ and Dectin-1- populations of myeloid cells in the TME and spleen of the CT26 tumor model. (**E**) Cumulative data comparing the percentages of I-A/I-E, CD80 and CD86 expressing cells among Dectin-1+ and Dectin-1- fractions of myeloid cells in the TME and spleen of CT26 and B16 tumor models. *P* values were calculated using One-way ANOVA (**A,B,D,E**).

Supplemental figure S4. Cumulative data of the MFI for Arg-I, ROS, TNF-α, IL-12, and Ki-67 in Dectin-1+ and Dectin-1- myeloid cells in B16 vs. CT26 tumor models. *P* values were calculated using One-way ANOVA.

Supplemental figure S5. (**A**) Cumulative data comparing percentages of PDL-1, PDL-2, VISTA, Tim-3 and Gal-9 expressing cells among Dectin-1+ and Dectin-1- myeloid subsets in B16 and CT26 tumor models. (**B**) Representative flow cytometry plots for co-expression of Dectin-1 with CD39, CD26 and CD73 in myeloid cells in the TME and Spleen of the B16 tumor model. (**C**) Cumulative data of percentages of CD39, CD26 and CD73 expressing cells among Dectin-1+ and Dectin-1- myeloid subsets in the spleen and TME of the B16 tumor model. (**D**) Representative Image cytometry plots showing the gating strategy for calculating co-localization of Dectin-1 and PDL-1/VISTA in myeloid cells in the TME. (**E**) Representative images of the H&E staining of a tumor tissue section. (**F**) Cumulative data of percentages of Dectin-1 expressing cells among CD3+, CD4+, and CD8+ T cells in the TME and spleen of B16 versus CT26 models. *P* values were calculated using two tailed, Mann-Whitney *t* test (**F**). One-way ANOVA (**A,C**).

Supplemental figure S6. (A) Representative histogram plots of GATA-3, RORγt and Tbet in Dectin-1- and Dectin-1+ subsets of T cells in in the TME and Spleen. (**B**) Cumulative data of the MFI of GATA-3, RORγt and Tbet in Dectin-1- and Dectin-1+ subsets of T cells in the TME and spleen. (**C**) Representative flow cytometry plots showing surface expression of CTLA-4, LAG-3, Tim-3, VISTA and Gal-9 on Dectin-1+ CD3+ and Dectin-1- CD3+ T cells in the TME. (**D**) Cumulative data of the MFI for CTLA-4, LAG-3, Tim-3, VISTA and Gal-9 on Dectin-1+ CD4+ and Dectin-1- CD4+ T cells in the TME. (**E**) Cumulative data of the MFI for CTLA-4, LAG-3, Tim-3, VISTA and Gal-9 on Dectin-1+ CD8+ and Dectin-1- CD8+ T cells in the TME. *P* values were calculated using two tailed, Mann-Whitney *t* test (**B,D,E**).

Supplemental figure S7. (**A**) Schematic picture of treatment schedule for WT tumor-bearing mice. (**B**) Representative images of spleens of WT, DKO and curdlan-treated WT tumor-bearing mice. (**C**) Cumulative data comparing the frequency of CD4+ T cells, CD8+ T cells and CD11b+ myeloid cells among WT, DKO and curdlan-treated WT tumor-bearing mice in the TME and spleen. (**D**) Cumulative data of the MFI of TNF-α expression in CD4+ T cells among WT, DKO and curdlan-treated WT tumor-bearing mice in the TME and spleen. (**E**) Cumulative data of the MFI of Gzm B and Perforin expression in CD8+ T cells among WT, DKO and curdlan-treated WT tumor-bearing mice in the TME and spleen. *P* values were calculated using One-way ANOVA (**C-E**).

Supplemental figure S8. (**A**) Schematic picture of treatment schedule for WT tumor-bearing mice with curdlan and α-VISTA (anti-VISTA) monoclonal antibody. (**B**) Representative images of spleens of WT, VISTA KO, curdlan-treated WT and curdlan plus α-VISTA treated WT tumor-bearing mice. (**C**) Cumulative data of the frequency of CD4+ T cells, CD8+ T cells, NK cells and CD11b+ myeloid cells among WT, VISTA KO, curdlan-treated WT and curdlan plus α-VISTA treated WT tumor-bearing mice. *P* values were calculated using One-way ANOVA (**C**).

Supplemental figure S9. (**A**) Cumulative data of the MFI of TNF-α and IL-12 in tumoral and splenic CD11b+ myeloid cells among WT, VISTA KO, curdlan-treated WT and curdlan plus α-VISTA treated WT tumor-bearing mice. (**B**) Cumulative data of the MFI for TNF-α and IFN-γ in tumoral and splenic CD11b+ myeloid cells among WT, VISTA KO, curdlan-treated WT and curdlan plus α-VISTA treated WT tumor-bearing mice. (**C**) Cumulative data comparing the MFI of GrzB and Perforin in tumoral and splenic CD8+ T cells among WT, VISTA KO, curdlan-

treated WT and curdlan plus α-VISTA treated WT tumor-bearing mice. (**D**) Schematic picture of treatment schedule for DKO tumor-bearing mice with α-VISTA monoclonal antibody. (**E**) Cumulative data of the frequency of CD8+ T cells among DKO and α-VISTA treated DKO tumor-bearing mice in the TME. (**F**) Cumulative data of the MFI for Gzm B and Perforin in tumoral and splenic CD8+ T cells among DKO and α-VISTA treated DKO tumor-bearing mice. *P* values were calculated using two tailed, Mann-Whitney *t* test (**E,F**). One-way ANOVA (**A-C**).

Supplemental figure S10. (**A**) Cumulative data comparing the MFI of TNF-α, IFN-γ and Ki-67 of tumoral CD11b+ myeloid cells among DKO and α-VISTA treated DKO tumor-bearing mice. (**B**) Cumulative data of the tumor size between VISTA KO and curdlan-treated VISTA KO tumor-bearing mice. (**C**) Representative images of dissected tumor tissues from VISTA KO and curdlan-treated VISTA KO tumor-bearing mice. (**D**) Cumulative data comparing spleen weight between VISTA KO and curdlan-treated VISTA KO tumor-bearing mice. (**E**) Representative images of spleens collected from VISTA KO and curdlan-treated VISTA KO tumor-bearing mice. (**F**) Cumulative data of the frequency of tumoral and splenic CD11b+ myeloid cells in VISTA KO and curdlan-treated VISTA KO tumor-bearing mice. (**G**) Cumulative data of the frequency of tumoral and splenic CD8+ T cells in VISTA KO and curdlan-treated VISTA KO tumor-bearing mice. (**H**) Cumulative data of the MFI for perforin and GrzB in tumoral and splenic CD8+ in VISTA KO and curdlan-treated VISTA KO tumor-bearing mice. (**I**) Representative pictures of dissected spleens from DKO tumor-bearing mice (B16-F10) treated or untreated with curdlan. (**J**) Heatmaps of RNAseq data of myeloid cells either treated or untreated with curdlan *in vitro*. *P* values were calculated using two tailed, Mann-Whitney *t* test (**A,B,D,F-H**).

Supplemental figure S11

The top 42 altered signaling pathways were selected with Ingenuity Pathway Analysis (IPA) software analysis. The bar chart represents activation and inhibition of signaling pathways using orange and blue bars, respectively, according to the z-score. The z-score indicates whether the direction of change is predicted to activate or inhibit the signaling pathways with a threshold set at a value of z-score >1.5.

Figure S1

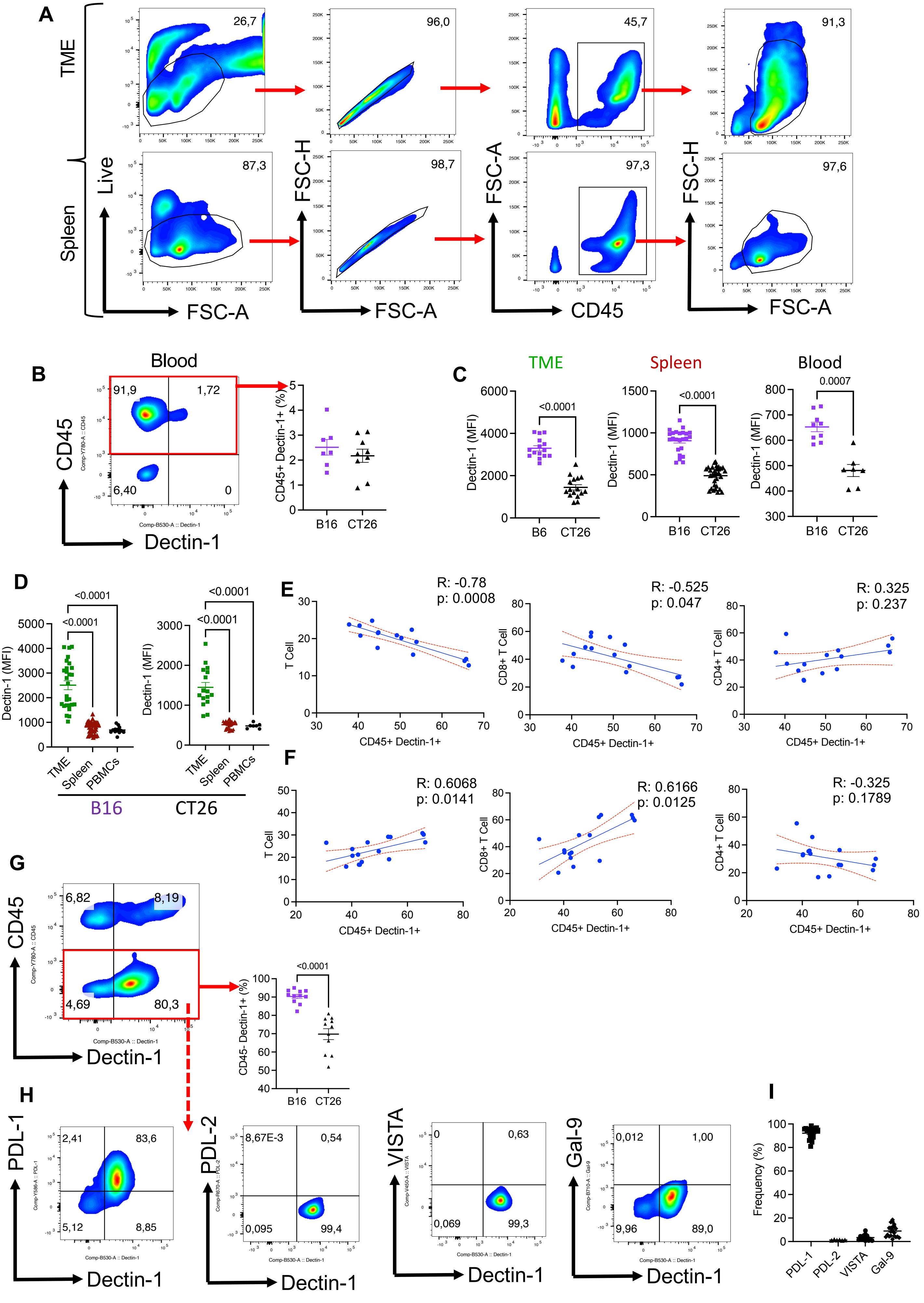
A
TME
Spleen
Live
FSC-A
FSC-H
FSC-A
FSC-A
CD45
FSC-H
FSC-A
26,7
96,0
45,7
91,3
87,3
98,7
97,3
97,6
B
Blood
CD45
Dectin-1
91,9
1,72
6,40
0
CD45+ Dectin-1+ (%)
B16
CT26
C
TME
Spleen
Blood
Dectin-1 (MFI)
<0.0001
<0.0001
0.0007
B6
CT26
B16
CT26
B16
CT26
D
Dectin-1 (MFI)
<0.0001
<0.0001
<0.0001
<0.0001
TME
Spleen
PBMCs
B16
CT26
E
R: -0.78
p: 0.0008
R: -0.525
p: 0.047
R: 0.325
p: 0.237
T Cell
CD8+ T Cell
CD4+ T Cell
CD45+ Dectin-1+
F
R: 0.6068
p: 0.0141
R: 0.6166
p: 0.0125
R: -0.325
p: 0.1789
T Cell
CD8+ T Cell
CD4+ T Cell
CD45+ Dectin-1+
G
CD45
Dectin-1
6,82
8,19
4,69
80,3
CD45- Dectin-1+ (%)
<0.0001
B16
CT26
H
PDL-1
2,41
83,6
5,12
8,85
PDL-2
8,67E-3
0,54
0,095
99,4
VISTA
0
0,63
0,069
99,3
Gal-9
0,012
1,00
9,96
89,0
Dectin-1
I
Frequency (%)
PDL-1
PDL-2
VISTA
Gal-9

Figure S2

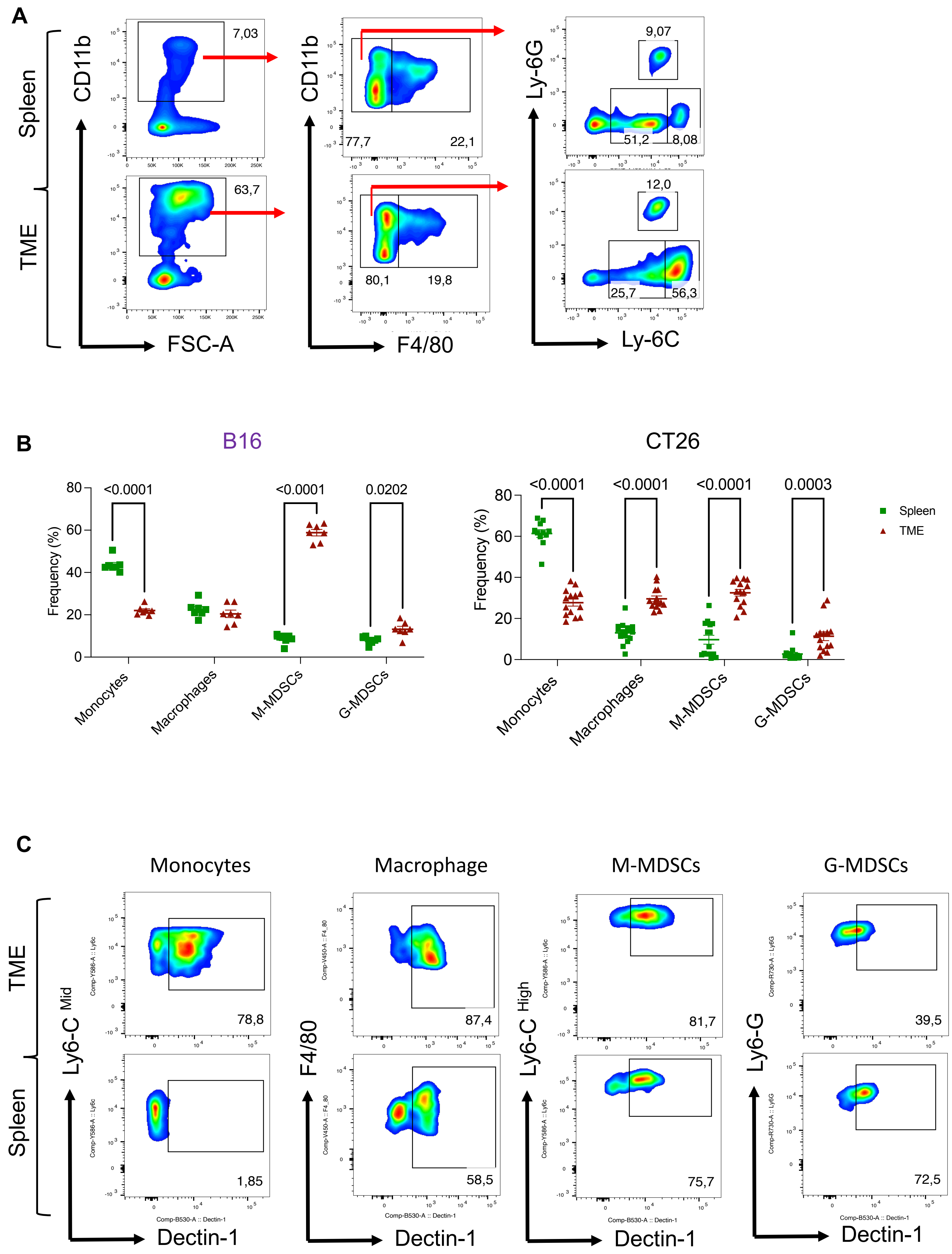
A
Spleen
TME
CD11b
FSC-A
7,03
63,7
F4/80
77,7
22,1
80,1
19,8
Ly-6G
Ly-6C
9,07
51,2
8,08
12,0
25,7
56,3
B
B16
CT26
Frequency (%)
<0.0001
0.0202
0.0003
Spleen
TME
Monocytes
Macrophages
M-MDSCs
G-MDSCs
C
Monocytes
Macrophage
M-MDSCs
G-MDSCs
TME
Spleen
Ly6-C Mid
F4/80
Ly6-C High
Ly6-G
Dectin-1
78,8
1,85
87,4
58,5
81,7
75,7
39,5
72,5

Figure S3

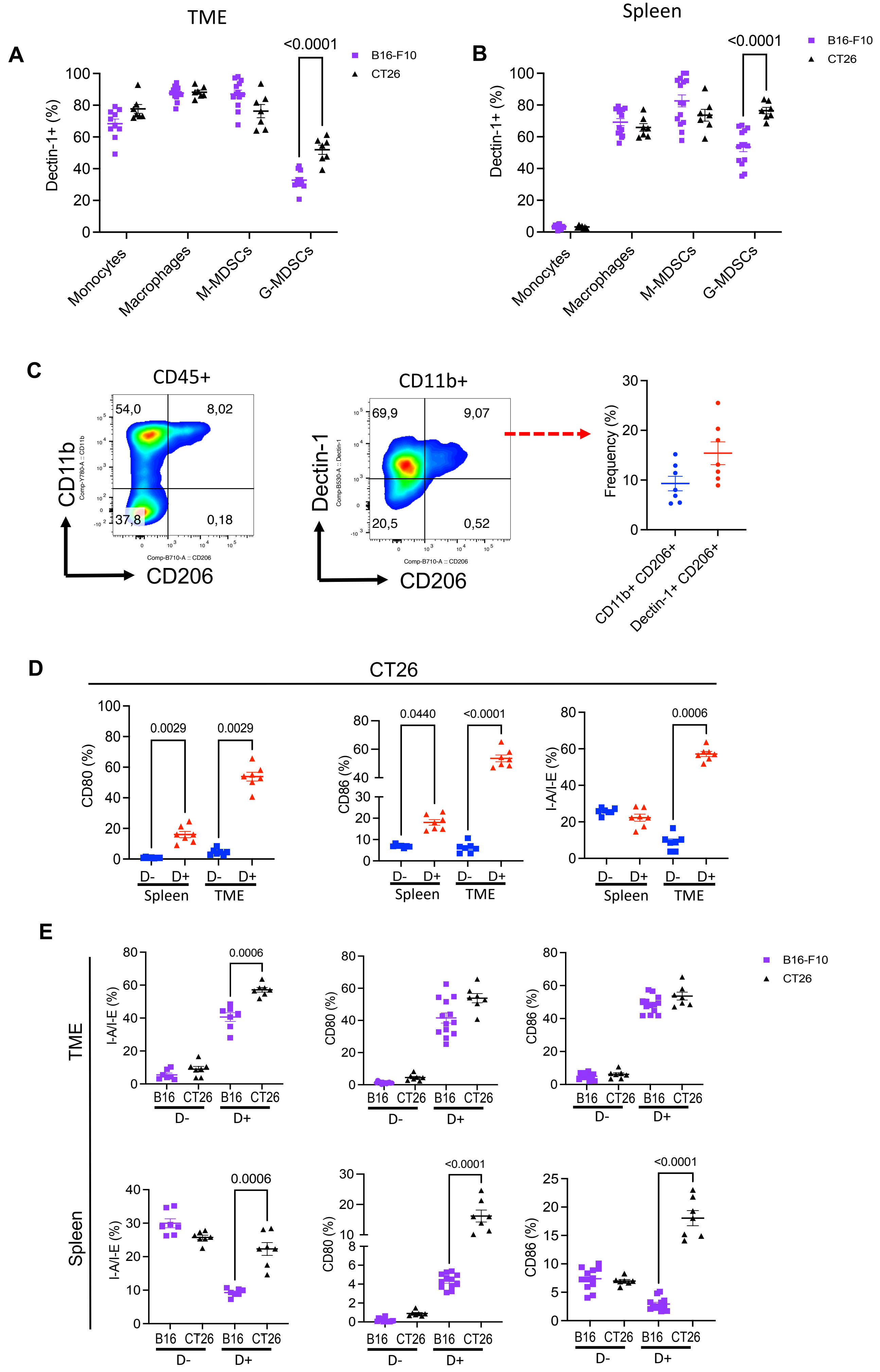

TME
Spleen
A
B
<0.0001
B16-F10
CT26
Dectin-1+ (%)
Monocytes
Macrophages
M-MDSCs
G-MDSCs
C
CD45+
CD11b+
54,0
8,02
37,8
0,18
69,9
9,07
20,5
0,52
CD11b
Dectin-1
CD206
Comp-B710-A :: CD206
Frequency (%)
CD11b+ CD206+
Dectin-1+ CD206+
D
CT26
0.0029
0.0029
CD80 (%)
0.0440
<0.0001
CD86 (%)
0.0006
I-A/I-E (%)
D-
D+
Spleen
TME
E
TME
Spleen
I-A/I-E (%)
CD80 (%)
CD86 (%)
0.0006
<0.0001
B16
CT26
D-
D+
B16-F10
CT26

Figure S4

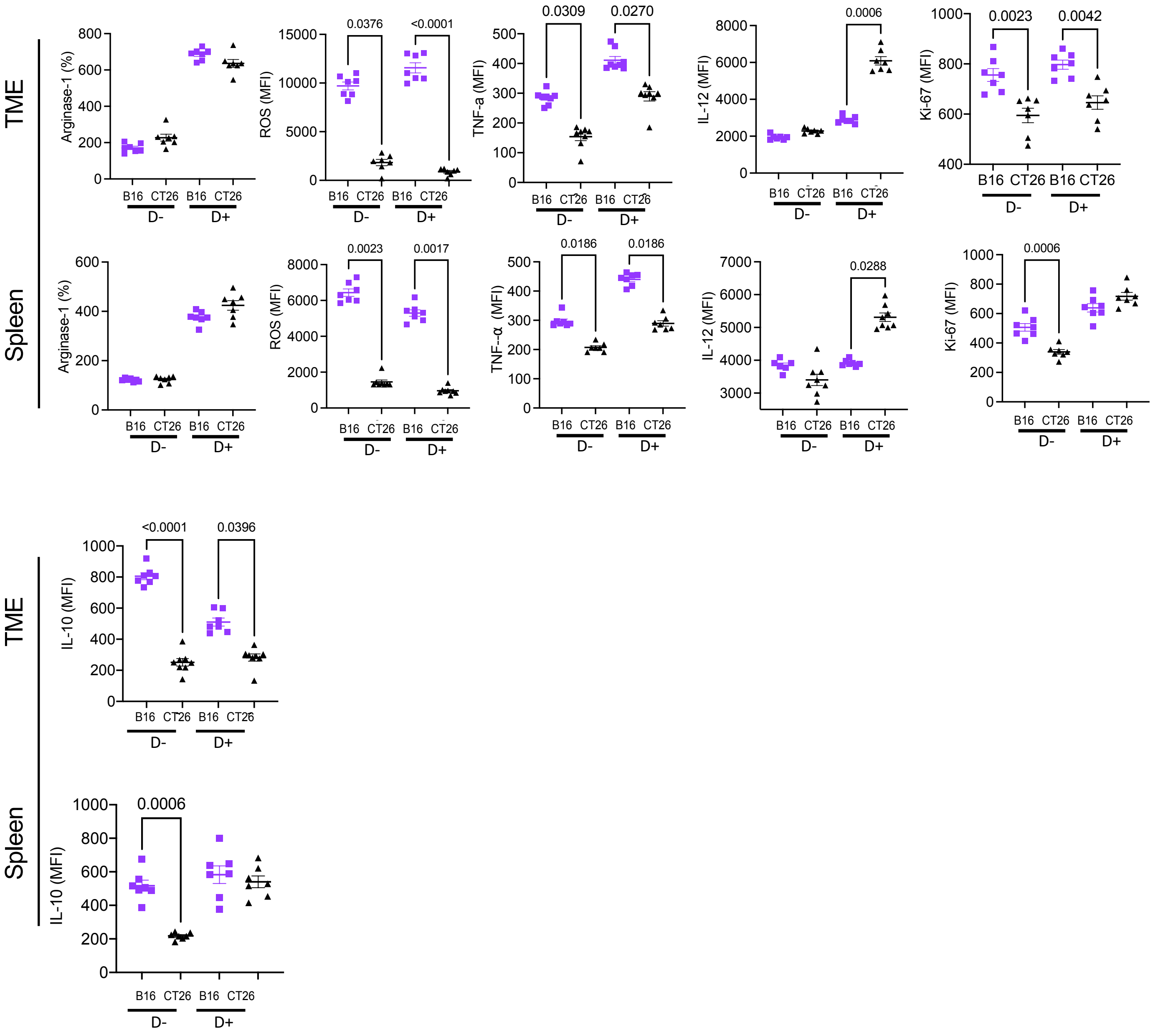
TME
Spleen
Arginase-1 (%)
ROS (MFI)
TNF-a (MFI)
IL-12 (MFI)
Ki-67 (MFI)
TNF--α (MFI)
IL-10 (MFI)
B16
CT26
D-
D+
0.0376
<0.0001
0.0309
0.0270
0.0006
0.0023
0.0042
0.0023
0.0017
0.0186
0.0186
0.0288
0.0006
<0.0001
0.0396
0.0006

Figure S5

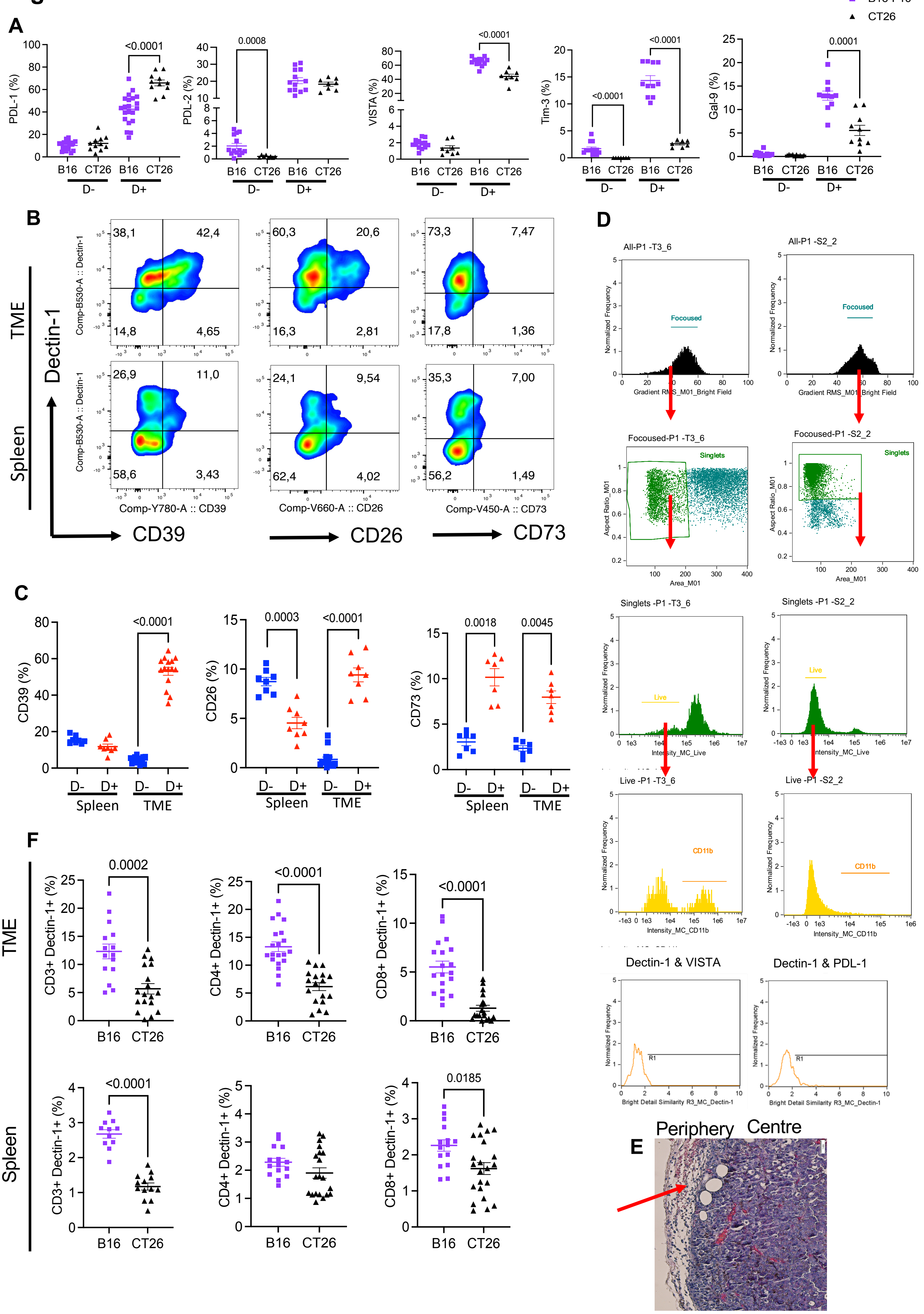

B16-F10
CT26
A
PDL-1 (%)
PDL-2 (%)
VISTA (%)
Tim-3 (%)
Gal-9 (%)
<0.0001
0.0008
<0.0001
<0.0001
<0.0001
0.0001
B16 CT26 B16 CT26
D- D+
B
TME
Spleen
Dectin-1
38,1 42,4 14,8 4,65
60,3 20,6 16,3 2,81
73,3 7,47 17,8 1,36
26,9 11,0 58,6 3,43
24,1 9,54 62,4 4,02
35,3 7,00 56,2 1,49
Comp-B530-A :: Dectin-1
Comp-Y780-A :: CD39
Comp-V660-A :: CD26
Comp-V450-A :: CD73
CD39
CD26
CD73
C
CD39 (%)
CD26 (%)
CD73 (%)
0.0003
0.0018
0.0045
D- D+ D- D+
Spleen TME
D
All-P1 -T3_6
All-P1 -S2_2
Focused
Gradient RMS_M01_Bright Field
Normalized Frequency
Focoused-P1 -T3_6
Focoused-P1 -S2_2
Singlets
Aspect Ratio_M01
Area_M01
Singlets -P1 -T3_6
Singlets -P1 -S2_2
Live
Intensity_MC_Live
Live -P1 -T3_6
Live -P1 -S2_2
CD11b
Intensity_MC_CD11b
Dectin-1 & VISTA
Dectin-1 & PDL-1
R1
Bright Detail Similarity R3_MC_Dectin-1
E
Periphery
Centre
F
CD3+ Dectin-1+ (%)
CD4+ Dectin-1+ (%)
CD8+ Dectin-1+ (%)
0.0002
0.0185
B16 CT26

Figure S6

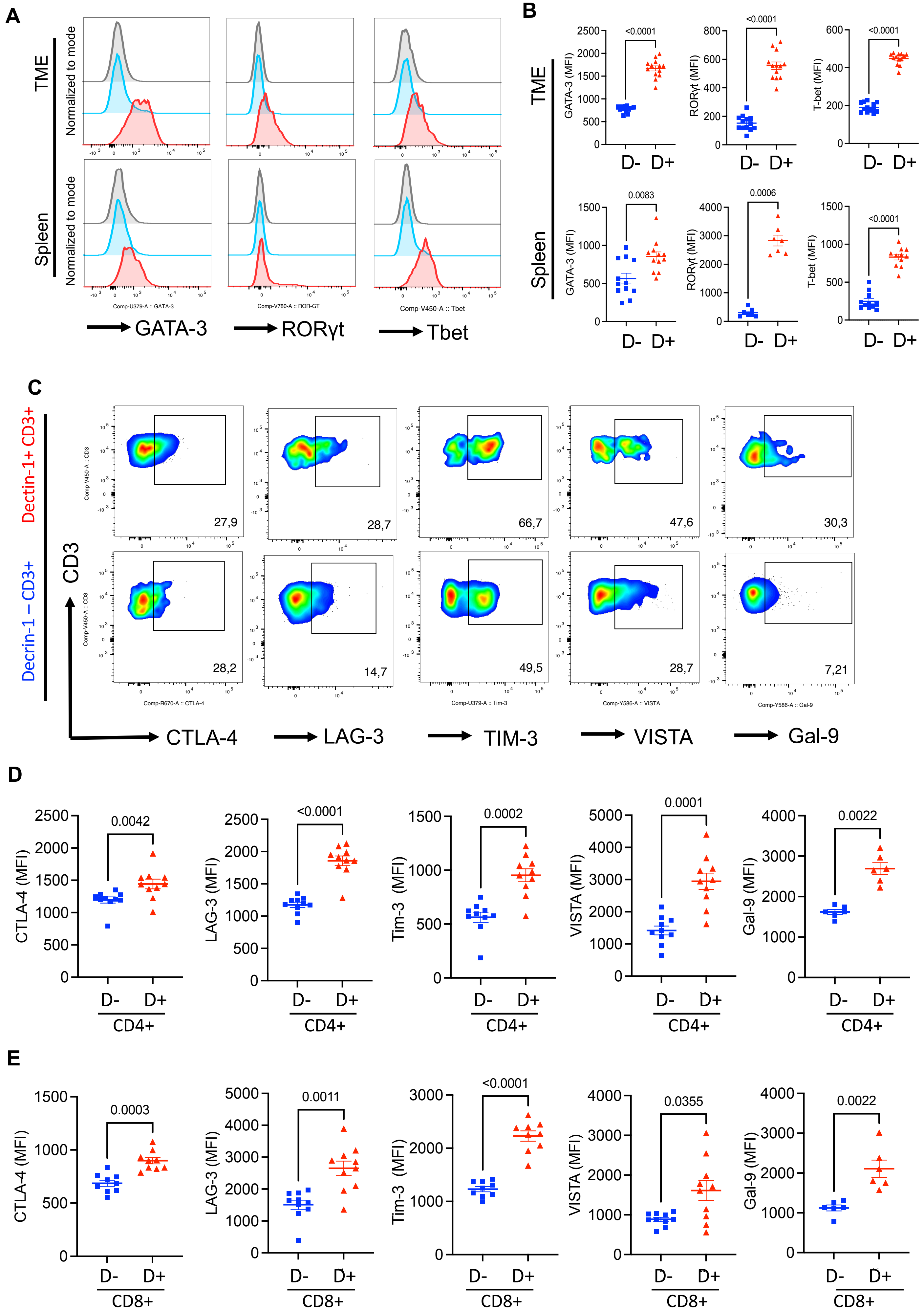

A
TME
Spleen
Normalized to mode
Comp-U379-A :: GATA-3
Comp-V780-A :: ROR-GT
Comp-V450-A :: Tbet
GATA-3
RORγt
Tbet
B
GATA-3 (MFI)
RORγt (MFI)
T-bet (MFI)
<0.0001
0.0083
0.0006
D- D+
C
Decin-1+ CD3+
Decrin-1 – CD3+
CD3
Comp-V450-A :: CD3
27,9
28,7
66,7
47,6
30,3
28,2
14,7
49,5
28,7
7,21
Comp-R670-A :: CTLA-4
Comp-U379-A :: Tim-3
Comp-Y586-A :: VISTA
Comp-Y586-A :: Gal-9
CTLA-4
LAG-3
TIM-3
VISTA
Gal-9
D
CTLA-4 (MFI)
LAG-3 (MFI)
Tim-3 (MFI)
VISTA (MFI)
Gal-9 (MFI)
0.0042
<0.0001
0.0002
0.0001
0.0022
CD4+
E
0.0003
0.0011
<0.0001
0.0355
0.0022
CD8+

Figure S7

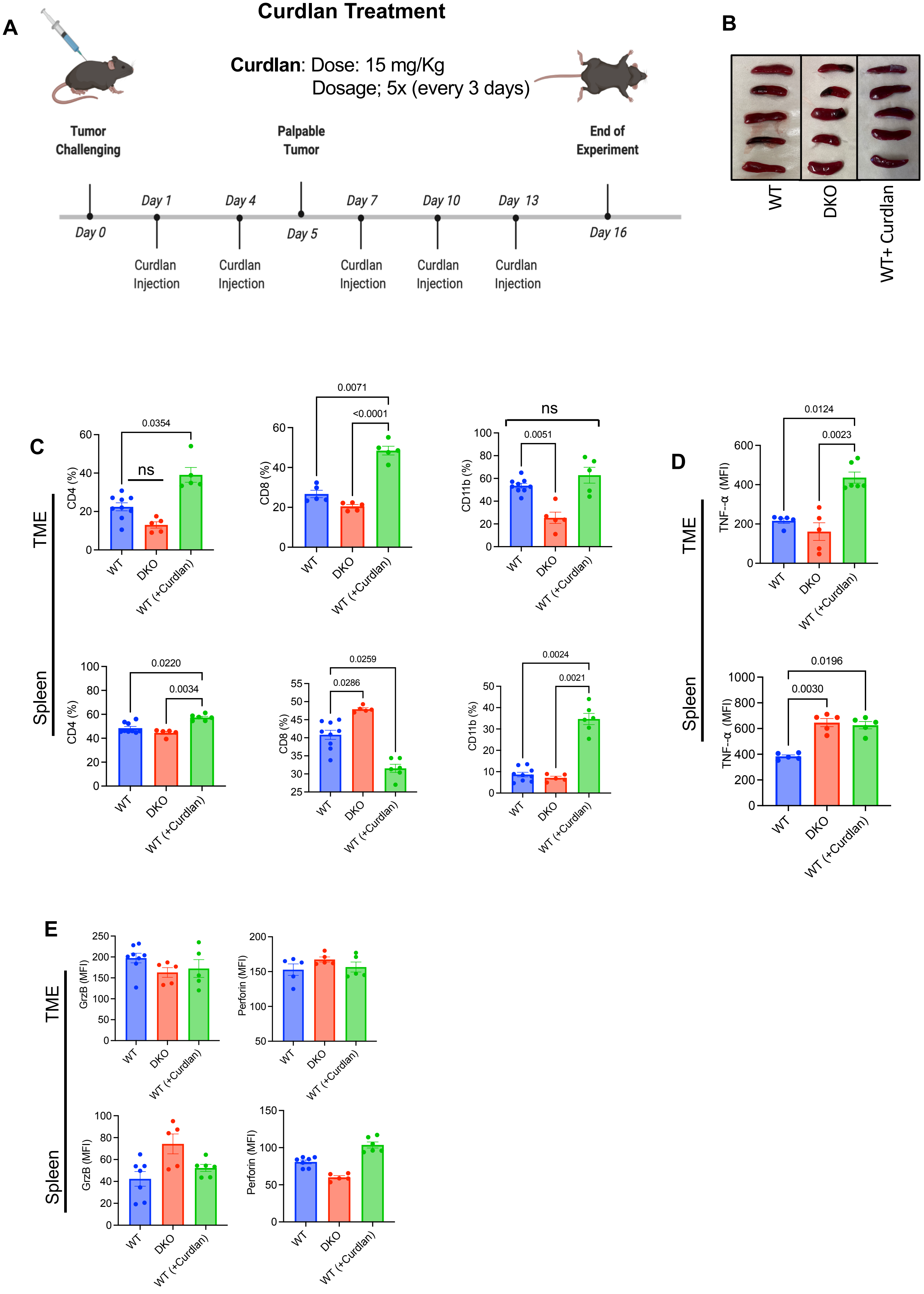
A
Curdlan Treatment
Curdlan: Dose: 15 mg/Kg
Dosage; 5x (every 3 days)
Tumor Challenging
Palpable Tumor
End of Experiment
Day 0
Day 1
Day 4
Day 5
Day 7
Day 10
Day 13
Day 16
Curdlan Injection
Curdlan Injection
Curdlan Injection
Curdlan Injection
Curdlan Injection
B
WT
DKO
WT+ Curdlan
C
TME
Spleen
CD4 (%)
0.0354
ns
CD8 (%)
0.0071
<0.0001
CD11b (%)
ns
0.0051
CD4 (%)
0.0220
0.0034
CD8 (%)
0.0259
0.0286
CD11b (%)
0.0024
0.0021
WT
DKO
WT (+Curdlan)
D
TME
Spleen
TNF--α (MFI)
0.0124
0.0023
TNF--α (MFI)
0.0196
0.0030
E
TME
Spleen
GrzB (MFI)
Perforin (MFI)
GrzB (MFI)
Perforin (MFI)

Figure S8

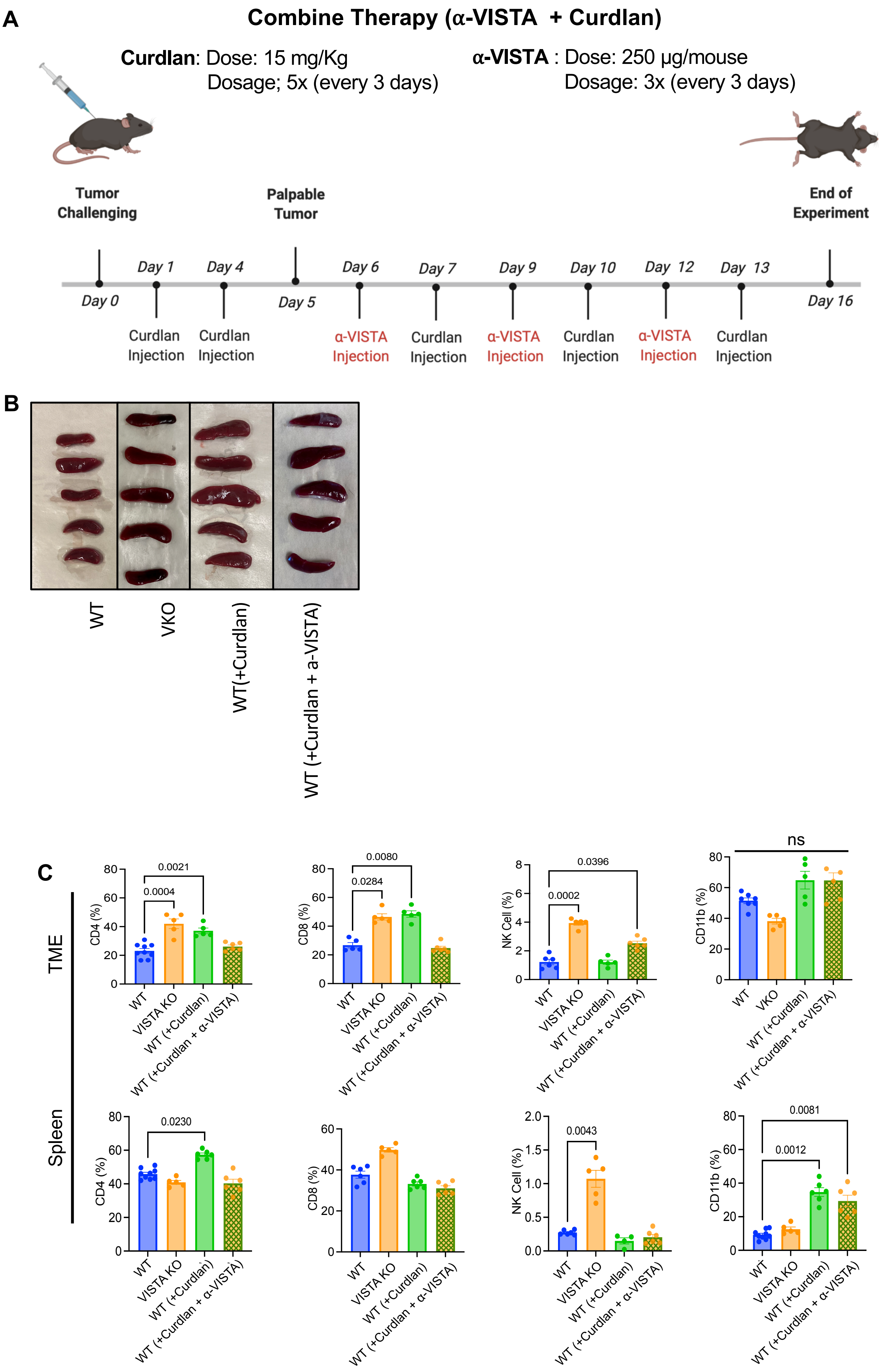

A
Combine Therapy (α-VISTA + Curdlan)
Curdlan: Dose: 15 mg/Kg
Dosage; 5x (every 3 days)
α-VISTA : Dose: 250 μg/mouse
Dosage: 3x (every 3 days)
Tumor Challenging
Palpable Tumor
End of Experiment
Day 0
Day 1
Day 4
Day 5
Day 6
Day 7
Day 9
Day 10
Day 12
Day 13
Day 16
Curdlan Injection
Curdlan Injection
α-VISTA Injection
Curdlan Injection
α-VISTA Injection
Curdlan Injection
α-VISTA Injection
Curdlan Injection
B
WT
VKO
WT(+Curdlan)
WT (+Curdlan + a-VISTA)
C
TME
Spleen
CD4 (%)
CD8 (%)
NK Cell (%)
CD11b (%)
0.0021
0.0004
0.0080
0.0284
0.0396
0.0002
ns
0.0230
0.0043
0.0081
0.0012
WT
VISTA KO
VKO
WT (+Curdlan)
WT (+Curdlan + α-VISTA)

Figure S9

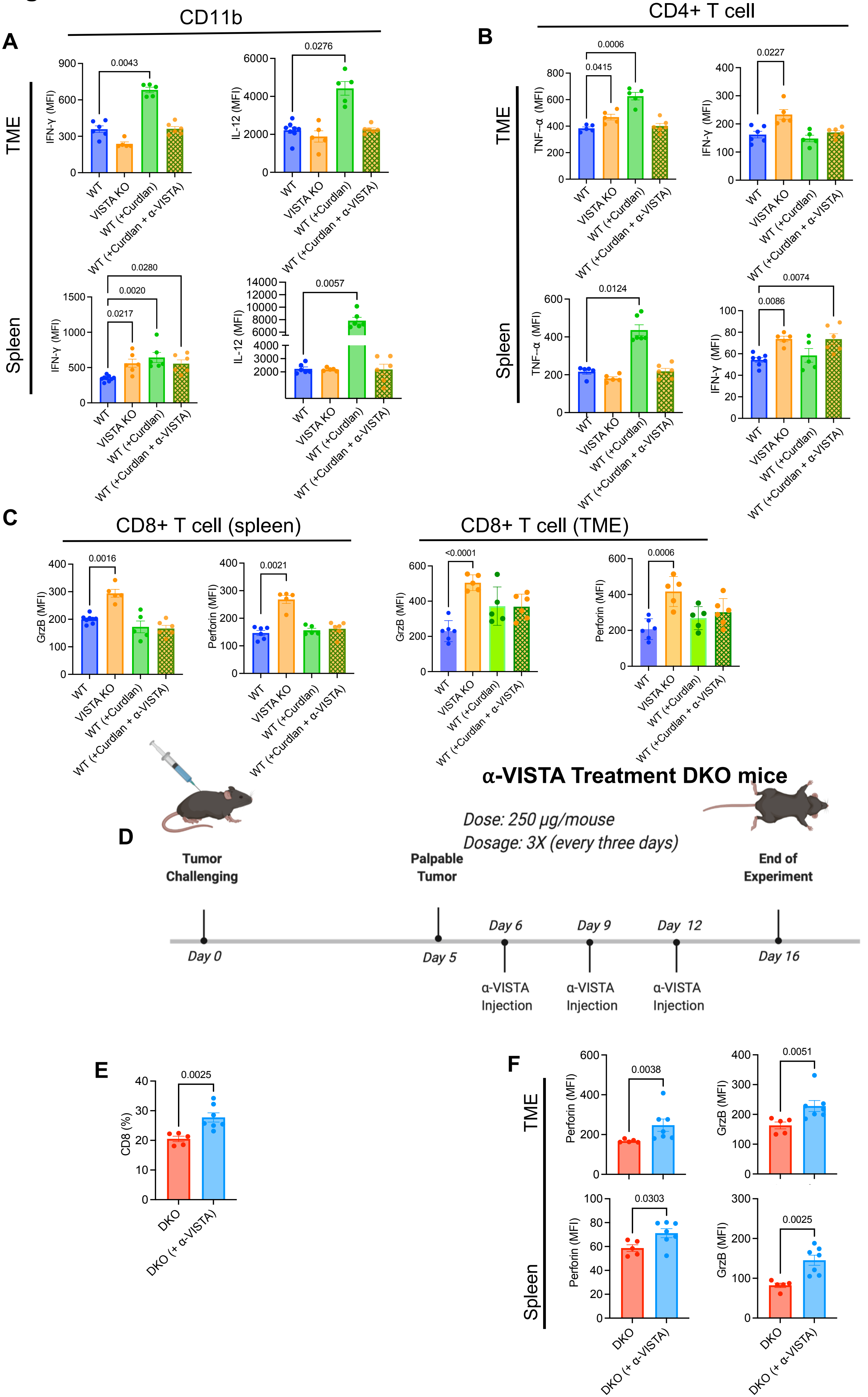

A
CD11b
TME
Spleen
IFN-γ (MFI)
IL-12 (MFI)
0.0043
0.0276
0.0280
0.0020
0.0217
0.0057
WT
VISTA KO
WT (+Curdlan)
WT (+Curdlan + α-VISTA)
B
CD4+ T cell
TNF-α (MFI)
0.0006
0.0415
0.0227
0.0124
0.0074
0.0086
C
CD8+ T cell (spleen)
CD8+ T cell (TME)
GrzB (MFI)
Perforin (MFI)
0.0016
0.0021
<0.0001
0.0006
α-VISTA Treatment DKO mice
Dose: 250 µg/mouse
Dosage: 3X (every three days)
D
Tumor Challenging
Palpable Tumor
End of Experiment
Day 0
Day 5
Day 6
Day 9
Day 12
Day 16
α-VISTA Injection
E
CD8 (%)
0.0025
DKO
DKO (+ α-VISTA)
F
0.0038
0.0051
0.0303
0.0025

Figure S10

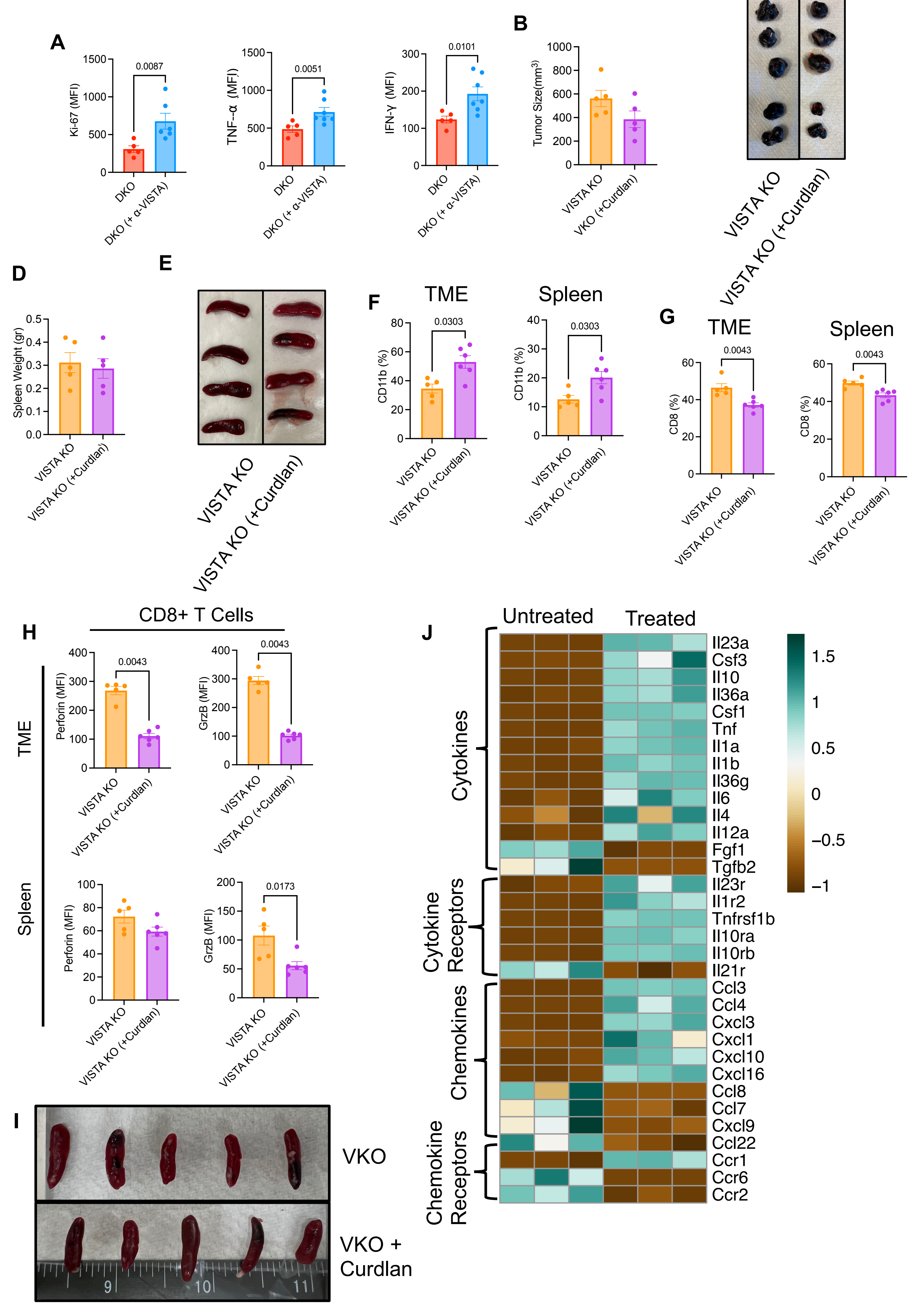

A
Ki-67 (MFI)
0.0087
DKO
DKO (+ α-VISTA)
TNF-α (MFI)
0.0051
IFN-γ (MFI)
0.0101
B
Tumor Size(mm3)
VISTA KO
VKO (+Curdlan)
C
VISTA KO
VISTA KO (+Curdlan)
D
Spleen Weight (gr)
E
F
TME
Spleen
CD11b (%)
0.0303
G
CD8 (%)
0.0043
H
CD8+ T Cells
TME
Spleen
Perforin (MFI)
GrzB (MFI)
0.0173
I
VKO
VKO + Curdlan
J
Untreated
Treated
Cytokines
Cytokine Receptors
Chemokines
Chemokine Receptors
Il23a
Csf3
Il10
Il36a
Csf1
Tnf
Il1a
Il1b
Il36g
Il6
Il4
Il12a
Fgf1
Tgfb2
Il23r
Il1r2
Tnfrsf1b
Il10ra
Il10rb
Il21r
Ccl3
Ccl4
Cxcl3
Cxcl1
Cxcl10
Cxcl16
Ccl8
Ccl7
Cxcl9
Ccl22
Ccr1
Ccr6
Ccr2

Figure S11

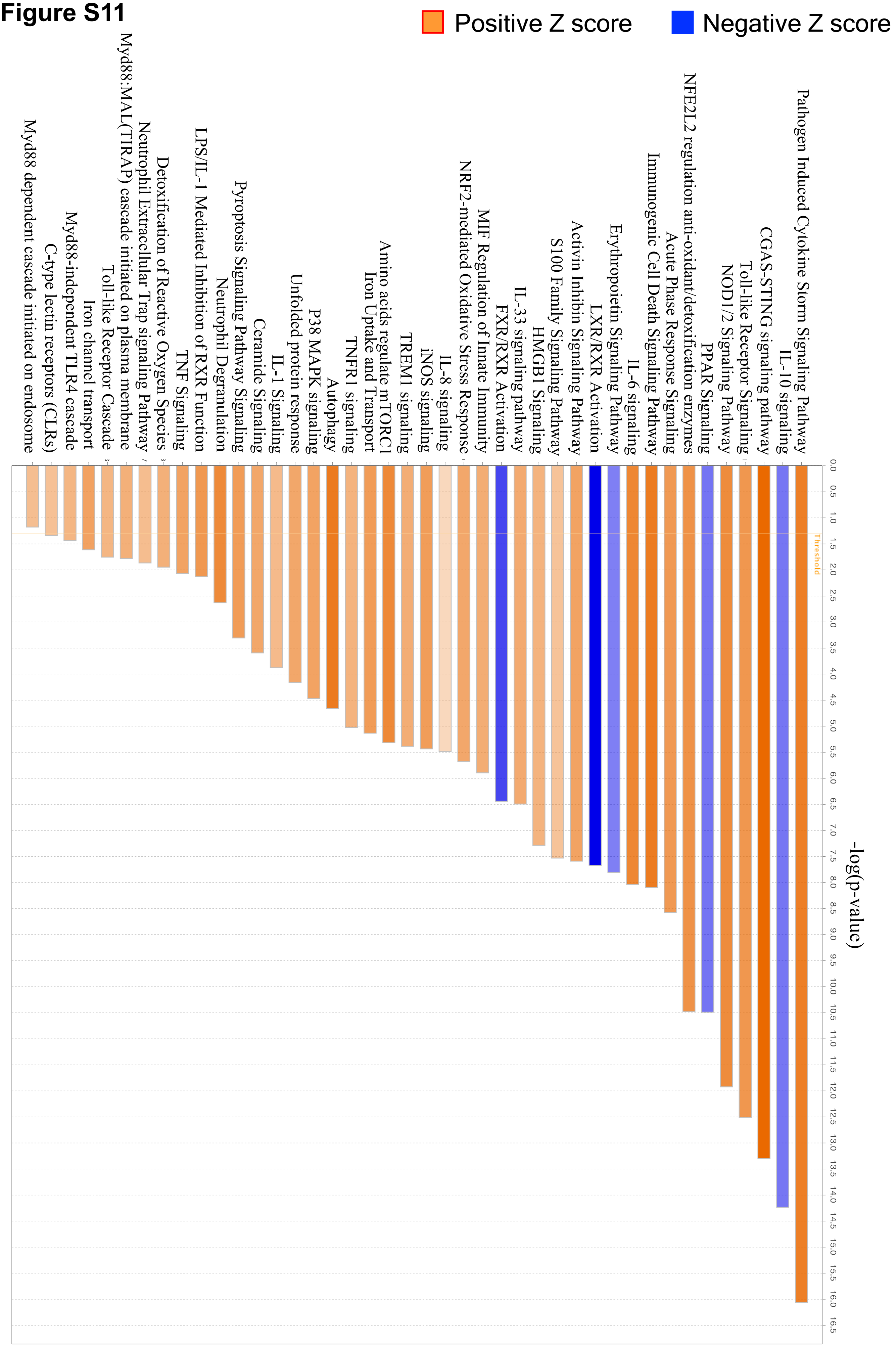

Positive Z score
Negative Z score
Pathogen Induced Cytokine Storm Signaling Pathway
IL-10 signaling
CGAS-STING signaling pathway
Toll-like Receptor Signaling
NOD1/2 Signaling Pathway
PPAR Signaling
NFE2L2 regulation anti-oxidant/detoxification enzymes
Acute Phase Response Signaling
Immunogenic Cell Death Signaling Pathway
IL-6 signaling
Erythropoietin Signaling Pathway
LXR/RXR Activation
Activin Inhibin Signaling Pathway
S100 Family Signaling Pathway
HMGB1 Signaling
IL-33 signaling pathway
FXR/RXR Activation
MIF Regulation of Innate Immunity
NRF2-mediated Oxidative Stress Response
IL-8 signaling
iNOS signaling
TREM1 signaling
Amino acids regulate mTORC1
Iron Uptake and Transport
TNFR1 signaling
Autophagy
P38 MAPK signaling
Unfolded protein response
IL-1 Signaling
Ceramide Signaling
Pyroptosis Signaling Pathway Signaling
Neutrophil Degranulation
LPS/IL-1 Mediated Inhibition of RXR Function
TNF Signaling
Detoxification of Reactive Oxygen Species
Neutrophil Extracellular Trap signaling Pathway
Myd88:MAL(TIRAP) cascade initiated on plasma membrane
Toll-like Receptor Cascade
Iron channel transport
Myd88-independent TLR4 cascade
C-type lectin receptors (CLRs)
Myd88 dependent cascade initiated on endosome
Threshold
-log(p-value)
