# Supplementary material for: Targeting Dectin-1 and or VISTA enhances anti-tumor immunity in melanoma but not colorectal cancer model
Source: Cell Oncol (Dordr). 2024 Apr 26;47(5):1735–56. doi: 10.1007/s13402-024-00950-w (PMC11467025; doi:10.1007/s13402-024-00950-w)
Supplement: Supplementary file 2 — Supplementary Material 2 [file 13402_2024_950_MOESM2_ESM.pdf]

Supplementary Table 1. The list of used antibodies and reagents in this study.

Antibodies/Reagents	Clone	Fluorophore	Catalog Number	Vendor
Monoclonal anti-mouse Arginase-1	IC5868A	APC	IC5868A	R&D
Monoclonal anti-mouse CD11b	M1/70	PE-Cy7	552850	BD Bioscience
Monoclonal anti-mouse CD11b	M1/70	PerCPCy5.5	550993	BD Bioscience
Monoclonal anti-mouse CD152 (CTLA-4)	UC10-4F10-11	APC	564331	BD Bioscience
Monoclonal anti-mouse CD206	MR6F3	PerCP-e710	46-2061-80	Invitrogen
Monoclonal anti-mouse CD223 (LAG-3)	C9B7W	BV786	740959	BD Bioscience
Monoclonal anti-mouse CD26	H194-112	BV650	740474	BD Bioscience
Monoclonal anti-mouse CD273 (PDL2)	TY25	APC	560086	BD Bioscience
Monoclonal anti-mouse CD274 (PDL1)	MIH5	PE	558091	BD Bioscience
Monoclonal anti-mouse CD3	17A2	APC-Cy7	560590	BD Bioscience
Monoclonal anti-mouse CD39	24DMS1	PE-Cy7	25-0391-82	eBioscience
Monoclonal anti-mouse CD3e	17A2	BV421	56-0032-80	eBioscience
Monoclonal anti-mouse CD4	GK1.5	BV786	563331	BD Bioscience
Monoclonal anti-mouse CD44	IM7	BV421	563970	BD Bioscience
Monoclonal anti-mouse CD45	30-F11	PE-Cy7	25045182	Invitrogen
Monoclonal anti-mouse CD45	30-F11	PE	553081	BD Bioscience
Monoclonal anti-mouse CD45	30-F11	PerCPCy5.5	550994	BD Bioscience
Monoclonal anti-mouse CD49d	R1-2	FITC	11-0492-82	Invitrogen
Monoclonal anti-mouse CD62L	MEL-14	PerCPCy5.5	560513	BD Bioscience
Monoclonal anti-mouse CD73	TY/23	v450	561544	BD Bioscience
Monoclonal anti-mouse CD8	53-6.7	A700	56-0081-82	eBioscience
Monoclonal anti-mouse CD80	16-10A1	e450	48-0801-82	eBioscience
Monoclonal anti-mouse CD86	GL1	PE	12-0862-82	eBioscience
Monoclonal anti-mouse Dectin-1 (CD369)	bg1fpj	PE-Cy7	25-5859-80	Invitrogen
Monoclonal anti-mouse Dectin-1 (CD369)	1,50E+03	FITC	MA5-16479	Invitrogen
Monoclonal anti-mouse F4/80	6F12	BV421	563900	BD Bioscience
Monoclonal anti-mouse Gal-9	RG9-35	PerCPCy5.5	136112	BioLegend
Monoclonal anti-mouse GATA3	L50-823	BUV395	565448	BD Bioscience
Monoclonal anti-mouse Granzyme B	NGZB	e450	48-8898-82	Invitrogen
Monoclonal anti-mouse I-A/I-E (MHC-II)	M5/114	PerCPCy5.5	562363	BD Bioscience
Monoclonal anti-mouse IFN-γ	XMG1.2	PE-Cy7	61-7311-82	eBioscience
Monoclonal anti-mouse IL-10	C17.8	PE	12-7101-82	eBioscience
Monoclonal anti-mouse IL12/IL-23p40	C17.8	e660	50-7123-82	eBioscience
Monoclonal anti-mouse Ki67	SolA15	e450	48-5698-82	eBioscience
Monoclonal anti-mouse Ly/6C	HK1.4	PE	12-5932-80	eBioscience
Monoclonal anti-mouse Ly/6G	1A8	Alexa 700	561236	BD Bioscience
Monoclonal anti-mouse NK1.1 (CD161)	PK136	PE	12-5941-82	eBioscience

Antibodies/Reagents	Clone	Fluorophore	Catalog Number	Vendor
Monoclonal anti-mouse Perforin	S16009B	PE	154405	BioLegend
Monoclonal anti-mouse RORγt	Q31-378	BV786	564723	BD Bioscience
Monoclonal anti-mouse Tbet	O4-46	V450	561312	BD Bioscience
Monoclonal anti-mouse Tim3 (CD366)	5D12	BUV395	747620	BD Bioscience
Monoclonal anti-mouse TNF-α	MP6-XT22	v450	560655	BD Bioscience
Monoclonal anti-mouse VISTA	MIH64	PE	566269	BD Bioscience
InVivoMAb anti-mouse VISTA	13F3	N/A	BE0310	BioXcell
Curdlan from Alcaligenes faecalis	N/A	N/A	C7821-5G	Sigma Aldrich
Fluorometric Intracellular Ros	N/A	FITC	MAK144-1KT	Sigma Aldrich
LIVE/DEAD™ Fixable Aqua Dead Cell Stain Kit	N/A	Aqua	L34966	ThermoFisher
